# Sitravatinib Sensitizes ABCB1- and ABCG2-Overexpressing Multidrug-Resistant Cancer Cells to Chemotherapeutic Drugs

**DOI:** 10.3390/cancers12010195

**Published:** 2020-01-13

**Authors:** Chung-Pu Wu, Sung-Han Hsiao, Yang-Hui Huang, Lang-Cheng Hung, Yi-Jou Yu, Yu-Tzu Chang, Tai-Ho Hung, Yu-Shan Wu

**Affiliations:** 1 Graduate Institute of Biomedical Sciences, College of Medicine, Chang Gung University, Tao-Yuan 33302, Taiwan; johnson170_ya@hotmail.com (S.-H.H.); yanghui.huang01@gmail.com (Y.-H.H.); ttpss91192@gmail.com (Y.-J.Y.); yyya.tw@yahoo.com.tw (Y.-T.C.); 2 Department of Physiology and Pharmacology, College of Medicine, Chang Gung University, Tao-Yuan 33302, Taiwan; 3 Department of Obstetrics and Gynecology, Taipei Chang Gung Memorial Hospital, Taipei 10507, Taiwan; thh20@adm.cgmh.org.tw; 4 Department of Chemistry, Tunghai University, Taichung 40704, Taiwan; oscarhung19000@gmail.com; 5 Department of Chinese Medicine, College of Medicine, Chang Gung University, Tao-Yuan 33302, Taiwan

**Keywords:** chemoresistance, MGCD516, modulators, P-glycoprotein, breast cancer resistance protein

## Abstract

The development of multidrug resistance (MDR) in cancer patients driven by the overexpression of ATP-binding cassette (ABC) transporter ABCB1 or ABCG2 in cancer cells presents one of the most daunting therapeutic complications for clinical scientists to resolve. Despite many novel therapeutic strategies that have been tested over the years, there is still no approved treatment for multidrug-resistant cancers to date. We have recently adopted a drug repurposing approach to identify therapeutic agents that are clinically active and at the same time, capable of reversing multidrug resistance mediated by ABCB1 and ABCG2. In the present study, we investigated the effect of sitravatinib, a novel multitargeted receptor tyrosine kinase inhibitor, on human ABCB1 and ABCG2 in multidrug-resistant cancer cell lines. We discovered that at submicromolar concentrations, sitravatinib re-sensitizes ABCB1- and ABCG2-overexpressing multidrug-resistant cancer cells to chemotherapeutic drugs. We found that sitravatinib blocks the drug efflux function of ABCB1 and ABCG2 in a concentration-dependent manner but does not significantly alter the protein expression of ABCB1 or ABCG2 in multidrug-resistant cancer cells. In conclusion, we reveal a potential drug repositioning treatment option for multidrug-resistant cancers by targeting ABCB1 and ABCG2 with sitravatinib and should be further investigated in future clinical trials.

## 1. Introduction

The development of multidrug resistance (MDR) in cancer cells, often caused by the overexpression of ATP-binding cassette (ABC) drug transporter, remains a major obstacle in cancer chemotherapy [1,2]. These multidrug resistance cancer cells are insensitive to a broad range of chemically unrelated anticancer drugs, which often led to treatment failure, relapse and eventual death of the patients [3,4]. Two of the most well-characterized, drug resistance linked ABC drug transporters are ABCB1 (MDR1; P-glycoprotein) and ABCG2 (BCRP; MXR) [1,2]. ABCB1 and ABCG2 are capable of utilizing energy derived from ATP hydrolysis to actively transport a large majority of conventional cytotoxic chemotherapeutic drugs including but not limited to, etoposide, anthracyclines, *Vinca alkaloids*, methotrexate, paclitaxel, topotecan, SN-38 and mitoxantrone, out of cancer cells and consequently reduced the intracellular accumulation of these drugs [2,5,6]. Clinically, high expression of these two transporters have been linked to the occurrence of multidrug resistance and poor clinical response in patients with metastatic breast cancer (MBC) [7], multiple myeloma (MM) [8,9,10], chronic lymphocytic leukaemia (CLL) [11], acute lymphocytic leukaemia (ALL) and acute myelogenous leukaemia (AML) [12,13,14]. Therefore, modulating the transport function or the protein expression of ABCB1 and/or ABCG2 is of great clinical significance.

To date, there is still no therapeutic agent approved by the U.S. Food and Drug Administration (FDA) for the modulation of ABCB1 or ABCG2 or treatment for patients with multidrug-resistant cancers. Numerous novel, potent synthetic inhibitors of ABCB1 and ABCG2 have been discovered over the years. However, factors associated with unfavourable drug-drug interactions and the lack of selectivity have hindered the further development of these inhibitors [5,15,16,17]. As an alternative, we and others have been investigating the potential repurposing of tyrosine kinase inhibitors (TKIs) as modulators of ABCB1 and ABCG2 to resensitize multidrug-resistant cancer cells to chemotherapeutic agents [18,19,20,21,22,23,24]. Sitravatinib (MGCD516) is a promising new receptor tyrosine kinase inhibitor [25] that is currently being evaluated in clinical trials for patients with MBC (ClinicalTrials.gov Identifier: NCT04123704), squamous cell carcinoma (SCC) (NCT03575598), advanced liposarcoma (NCT02978859), advanced urothelial carcinoma (NCT03606174), advanced clear cell renal cell carcinoma (NCT03680521), non-small cell lung cancer (NSCLC) (NCT02664935), advanced non-squamous NSCLC (NCT03906071), advanced solid tumour malignancies (NCT02219711 and NCT03666143), advanced kidney cancer (NCT03015740) and unresectable locally advanced hepatocellular carcinoma (HCC) or gastric/gastroesophageal junction cancer (GC/GEJC) (NCT03941873). At this time, the relationship between sitravatinib and ABCB1 or ABCG2 remains elusive.

In the present work, we discovered an additional action of sitravatinib in human cancer cells. We demonstrated that by modulating the transport function of both ABCB1 and ABCG2, sitravatinib re-sensitizes ABCB1- and ABCG2-overexpressing multidrug-resistant cancer cells to chemotherapeutic agents.

## 2. Results

### 2.1. Drug-Sensitive Cancer Cells and Multidrug-Resistant Cancer Cells Overexpressing ABCB1 or ABCG2 Are Equally Sensitive to Sitravatinib

**Figure 1:**
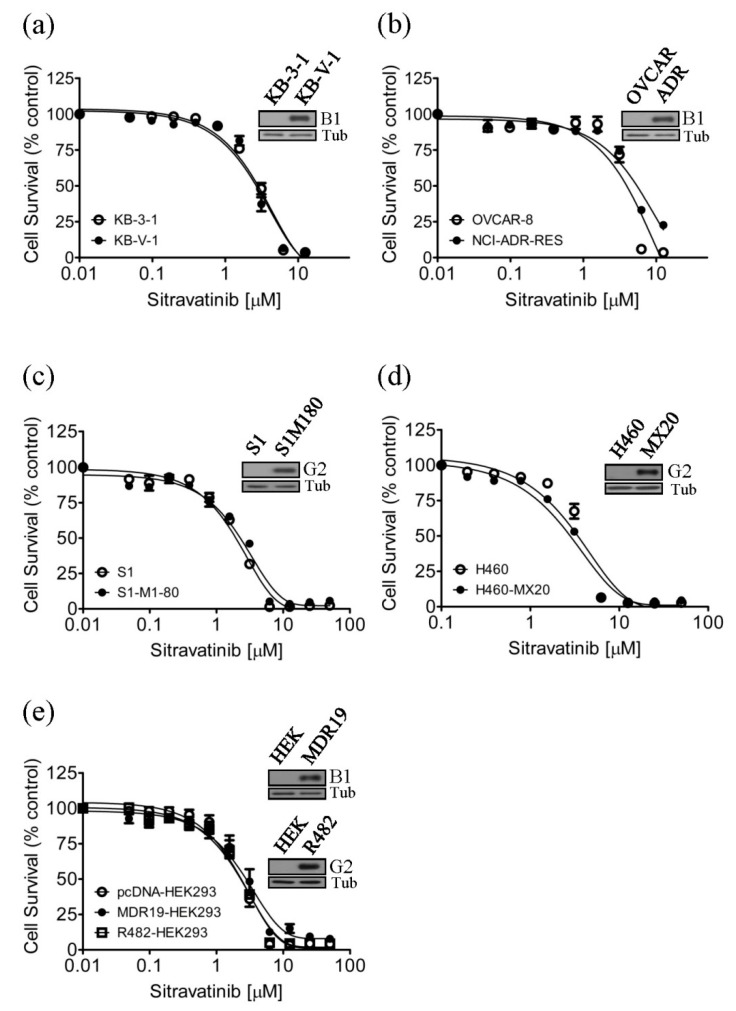
Drug-sensitive cells and multidrug-resistant cells overexpressing ABCB1 or ABCG2 are equally sensitive to sitravatinib. The cytotoxicity of sitravatinib was determined in (**a**) drug-sensitive parental KB-3-1 human epidermal cancer cells (open circles) and the ABCB1-overexpressing MDR variant KB-V-1 (filled circles), (**b**) drug-sensitive parental OVCAR-8 human ovarian cancer cells (open circles) and the ABCB1-overexpressing MDR variant NCI-ADR-RES (filled circles), (**c**) drug-sensitive parental S1 human colon cancer cells (open circles) and the ABCG2-overexpressing MDR variant S1-M1-80 (filled circles), (**d**) drug-sensitive H460 human lung cancer cells (open circles) and the ABCG2-overexpressing MDR variant H460-MX20 (filled circles), as well as (**e**) parental pcDNA-HEK293 cells (open circles) and HEK293 cells transfected with human ABCB1 (MDR19-HEK293, filled circles) or human ABCG2 (R482-HEK293, open squares). Representative immunoblots of ABCB1 in KB-3-1 and KB-V-1 ((**a**), inset), in OVCAR-8 and NCI-ADR-RES ((**b**), inset) and in HEK293 and MDR19-HEK293 ((**e**), inset), as well as ABCG2 in S1 and S1-M1-80 ((**c**), inset), in H460 and H460-MX20 ((**d**), inset) and in HEK293 and R482-HEK293 ((**e**), inset). Measuring points, mean values from at least three independent experiments; bars; standard error of the mean (S.E.M).

Previous studies have reported that tyrosine kinase inhibitors (TKIs) such as imatinib [26,27,28,29] and sunitinib [30,31] are transported substrates of ABCB1 and ABCG2 and the overexpression of ABCB1 or ABCG2 in cancer cells results in reduced susceptibility to TKIs [32,33,34]. To this end, we examined whether cells overexpressing ABCB1 or ABCG2 are less sensitive to sitravatinib than the drug-sensitive counterpart cells. We found that the ABCB1-overexpressing human KB-V-1 epidermal cancer cells (Figure 1a) and the ABCB1-overexpressing human NCI-ADR-RES ovarian cancer cells (Figure 1b) are equally sensitive to sitravatinib as their drug-sensitive parental KB-3-1 and OVCAR-8 cancer cells, respectively. Similarly, we found that the ABCG2-overexpressing human S1-M1-80 colon cancer cells (Figure 1c) and the ABCG2-overexpressing human H460-MX20 lung cancer cells (Figure 1d) are equally sensitive to sitravatinib as their parental S1 and H460 cancer cells, respectively. To further confirm our findings, we compared the cytotoxicity of sitravatinib in HEK293 cells and HEK293 cells transfected with human ABCB1 (MDR19-HEK293) or human ABCG2 (R482-HEK293). As shown in Figure 1e, these HEK293 cell lines are equally sensitive to sitravatinib treatment. The IC_50_ value of sitravatinib in tested cell lines are summarized in Table 1 and the resistance factor (R.F) value, signifying the degree of resistance caused by the overexpression of ABCB1 or ABCG2, was calculated by dividing the IC_50_ value of sitravatinib in ABCB1- or ABCG2-overexpressing cell lines by the IC_50_ value of sitravatinib in respective parental cell lines.

### 2.2. Sitravatinib Reverses Multidrug Resistance Mediated by ABCB1 and ABCG2

**Figure 2:**
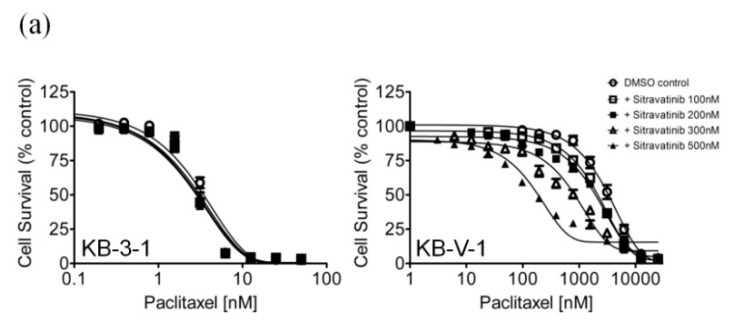

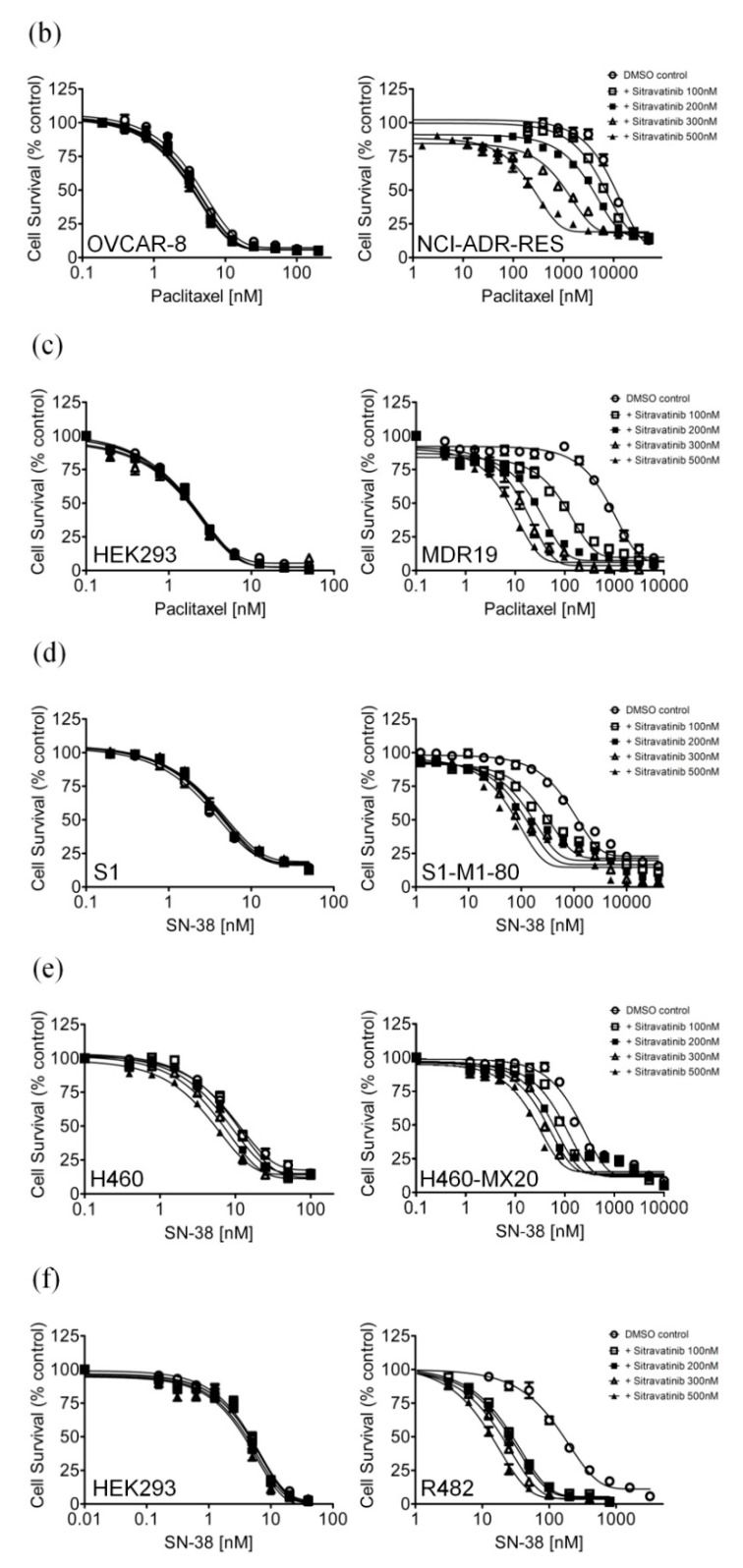
Sitravatinib re-sensitizes ABCB1-overexpressing cells to paclitaxel and ABCG2-overexpressing cells to SN-38. The chemosensitization effect of sitravatinib on ABCB1-overexpressing or ABCG2-overexpressing multidrug-resistant cells was examined by treating (**a**) KB-3-1 and ABCB1-overexpressing KB-V-1 cells, (**b**) OVCAR-8 and ABCB1-overexpressing NCI-ADR-RES cells and (**c**) pcDNA-HEK293 and ABCB1-transfected MDR19-HEK293 cells with increasing concentrations of paclitaxel, a known substrate of ABCB1 or treating (**d**) S1 and ABCG2-overexpressing S1-M1-80 cells, (**e**) H460 and ABCG2-overexpressing H460-MX20 and (**f**) pcDNA-HEK293 and ABCG2-transfected R482-HEK293 cells with increasing concentrations of SN-38, a known substrate of ABCG2. Cells were treated with paclitaxel or SN-38 in the presence of dimethyl sulfoxide (DMSO control, open circles) or sitravatinib at 100 nM (open squares) or sitravatinib at 200 nM (filled squares) or sitravatinib at 300 nM (open triangles) or sitravatinib at 500 nM (filled triangles). Measuring points, mean values from at least three independent experiments; bars; S.E.M.

Notably, previous studies have reported that certain TKIs can reverse multidrug resistance mediated by ABCB1 and ABCG2 in cancer cells by interacting strongly with these ABC drug transporters [24,35,36]. To this end, we examined the potential chemosensitization effect of sitravatinib in cell lines overexpressing ABCB1 or ABCG2. We discovered that at sub-toxic concentrations, sitravatinib did not significantly affect the drug-sensitive parental cells but re-sensitized the ABCB1-overexpressing KB-V-1 (Figure 2a), NCI-ADR-RES (Figure 2b) and the ABCB1-transfected MDR19-HEK293 cells (Figure 2c) to paclitaxel, a known substrate of ABCB1 [37], in a concentration-dependent manner. Moreover, we found that sitravatinib re-sensitized ABCB1-overexpressing cells to other ABCB1 substrates, colchicine and vincristine [37,38], in the same manner (Table 2). Of note, verapamil at 5 μM was used here as a reference inhibitor for ABCB1. Furthermore, we found that ABCG2-mediated resistance to SN-38, a known substrate of ABCG2 [39], was reversed by sitravatinib in ABCG2-overexpressing S1-M1-80 (Figure 2d), H460-MX20 (Figure 2e) and ABCG2-transfected R482-HEK293 cells (Figure 2f) in a concentration-dependent manner. As shown in Table 3, we demonstrated that sitravatinib resensitized ABCG2-overexpressing cells to ABCG2 substrates, mitoxantrone and topotecan [40,41], in a similar manner. Notably, Ko143 at 1 μM was used here as a reference inhibitor for ABCG2. As shown in Table 2 and Table 3, the fold-reversal (FR) value, signifying the extent of resensitization by a particular compound in tested cell lines, was calculated by dividing the IC_50_ value of the drug substrate alone by the IC_50_ value of the drug substrate in the presence of sitravatinib or a reference inhibitor, as described previously [42]. Our results showed that sitravatinib reverses multidrug resistance mediated by ABCB1 and ABCG2 in a concentration-dependent manner and at submicromolar concentrations.

### 2.3. Sitravatinib Restores Sensitivity for Drug-Induced Apoptosis in ABCB1- and ABCG2-Overexpressing Multidrug-Resistant Cancer Cells

**Figure 3:**
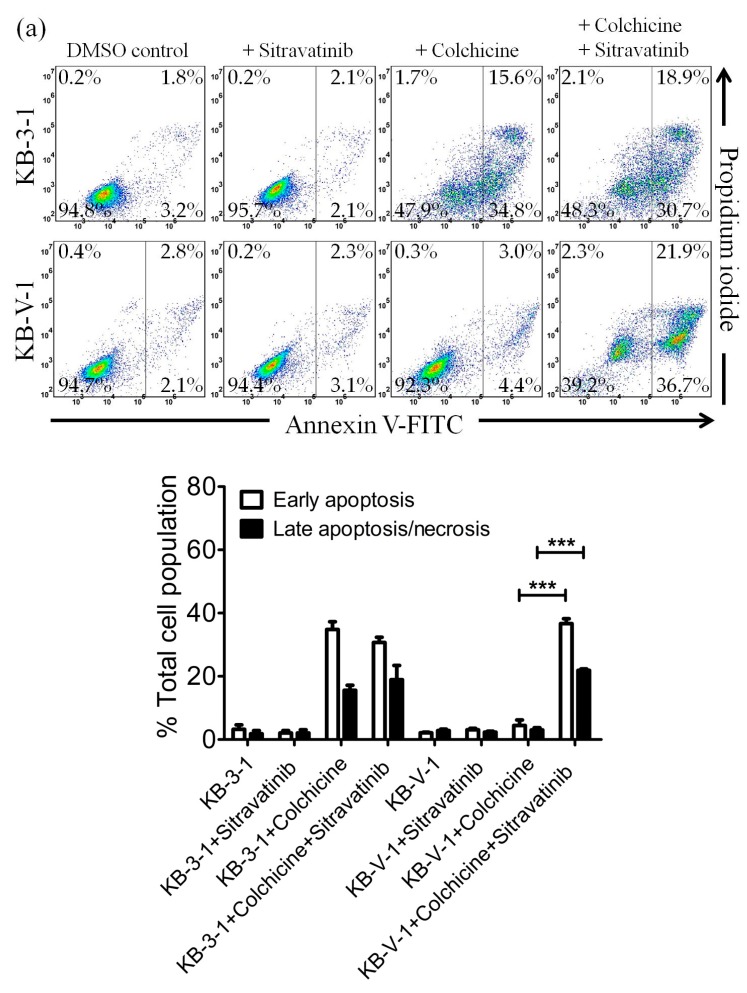

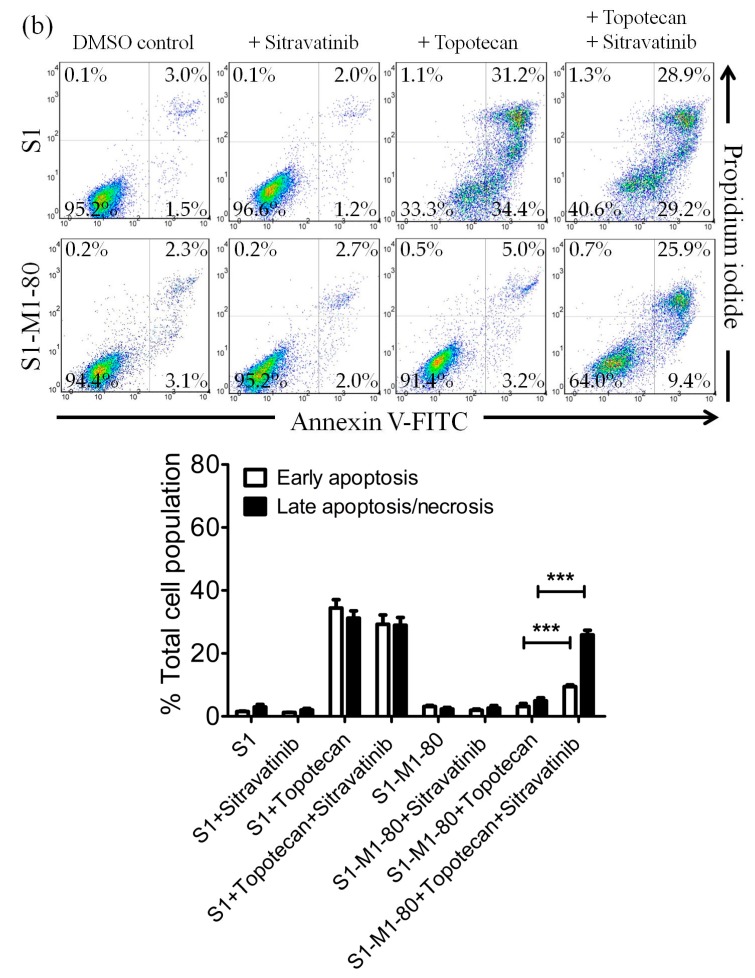
Sitravatinib restores apoptosis sensitivity in multidrug-resistant cancer cells overexpressing ABCB1 or ABCG2. Dot plots (upper panel) and quantification (lower panel) of (**a**) drug-sensitive parental KB-3-1 and the ABCB1-overexpressing subline KB-V-1treated with either DMSO (control), 5 μM of sitravatinib (+sitravatinib), 500 nM of colchicine (+colchicine) or a combination of 500 nM of colchicine and 5 μM of sitravatinib (+colchicine +sitravatinib) and (**b**) drug-sensitive parental S1 and the ABCG2-overexpressing subline S1-M1-80 treated with either DMSO (control), 5 μM of sitravatinib (+sitravatinib), 5 μM of topotecan (+topotecan) or a combination of 5 μM of topotecan and 5 μM of sitravatinib (+topotecan + sitravatinib). Cells were treated with respective regimens, isolated and analysed by flow cytometry as described previously [44]. Representative dot plots and quantifications of apoptotic cell populations are presented as mean ± SD calculated from at least three independent experiments are shown. *** *p* < 0.001, versus the same treatment in the absence of sitravatinib.

In order to distinguish the potential growth retardation effect from the drug-induced cytotoxicity restored by sitravatinib, we examined the effect of sitravatinib on apoptosis induced by colchicine or topotecan, both known inducers of apoptosis [38,43], in ABCB1-overexpressing KB-V-1 cancer cells and ABCG2-overexpressing S1-M1-80 cancer cells, respectively. Drug-sensitive parental cancer cells and multidrug-resistant cancer cells were treated with the indicated drug regimens as detailed in Materials and Methods. As shown in Figure 3a, while 500 nM of colchicine induced substantial apoptosis in KB-3-1 cells (from approximately 5% basal level to 50% of early and late apoptosis), it had minimal effect on the level of apoptosis in ABCB1-overexpressing KB-V-1 cells (from approximately 5% basal level to 7% of early and late apoptosis) for colchicine, a transported substrate of ABCB1 [37]. We found that treatment with sitravatinib alone did not induce apoptosis in KB-3-1 or KB-V-1 cells but it significantly increased the colchicine induced apoptosis in KB-V-1 cells, from 7% to 58 % of total apoptosis. Moreover, as shown in Figure 3b, when treated with 5 μM of topotecan, the proportion of apoptotic cells increased significantly, from approximately 5% basal level to 58% of early and late apoptosis in S1 cells but not in ABCG2-overexpressing S1-M1-80 cells due to ABCG2-mediated efflux of topotecan. Although treatment with sitravatinib alone did not substantially induce apoptosis in S1 or S1-M1-80 cells, it significantly enhanced the topotecan-induced apoptosis in S1-M1-80 cells, from 8% to 35% of early and late apoptosis.

### 2.4. Sitravatinib Increases Drug Accumulation in Cells Overexpressing ABCB1 or ABCG2

**Figure 4:**
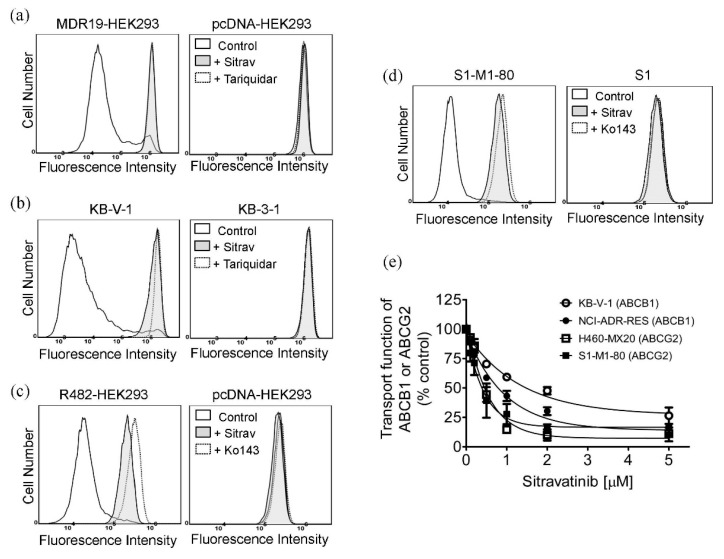
Sitravatinib increases the intracellular accumulation of fluorescent substrates of ABCB1 and ABCG2 by modulating the drug efflux function of ABCB1 and ABCG2. The intracellular accumulation of fluorescent ABCB1 substrate calcein was determined in (**a**) ABCB1-transfected MDR19-HEK293 cells (left panel) and (**b**) ABCB1-overexpressing KB-V-1 cancer cells (left panel) in the presence of DMSO (control, solid line), 5 μM of sitravatinib (+Sitrav, filled solid line) or 3 μM of tariquidar (+Tariquidar, dotted line) as a positive control for ABCB1 inhibition, whereas the intracellular accumulation of fluorescent ABCG2 substrate pheophorbide A (PhA) was determined in (**c**) ABCG2-transfected R482-HEK293 (left panel) and (**d**) ABCG2-overexpressing S1-M1-80 cancer cells (left panel) in the presence of DMSO (control, solid line), 5 μM of sitravatinib (+Sitrav, filled solid line) or 1 μM of Ko143 (+Ko143, dotted line) as a positive control for ABCG2 inhibition. Respective drug-sensitive parental cells were treated with identical conditions and used as controls ((**a**–**d**), right panels). The respective fluorescent signals were analysed by flow cytometry as described in Materials and Methods. Representative histograms of at least three independent experiments are shown. (**e**) Concentration-dependent inhibition of ABCB1-mediated calcein efflux and ABCG2-mediated PhA efflux in KB-V-1 cancer cells (open circles), NCI-ADR-RES cancer cells (filled circles), H460-MX20 cancer cells (open squares) and S1-M1-80 cancer cells (filled squares). Measuring points, mean values from at least three independent experiments; bars; S.E.M.

Next, we examined the effect of sitravatinib on the drug transport function of both ABCB1 and ABCG2 by monitoring the intracellular accumulation of fluorescent drug substrates of ABCB1 and ABCG2 in the absence and presence of sitravatinib in cells overexpressing ABCB1 or ABCG2. We found that 5 μM of sitravatinib was able to block the drug transport function of ABCB1 and significantly increased the intracellular accumulation of calcein, a fluorescent product of an ABCB1 substrate calcein-AM [45], in ABCB1-transfected MDR19-HEK293 cells (Figure 4a) and ABCB1-overexpressing KB-V-1 cancer cells (Figure 4b). Similarly, 5 μM of sitravatinib also increases the accumulation of pheophorbide A (PhA), a fluorescent substrate of ABCG2 [46], in ABCG2-transfected R482-HEK293 cells (Figure 4c) and ABCG2-overexpressing S1-M1-80 cancer cells (Figure 4d). Of note, sitravatinib did not have a significant effect on the accumulation of fluorescent drugs in drug-sensitive parental cell lines (Figure 4a–d, right panels). Tariquidar (3 μM) and Ko143 (1 μM) were used as reference inhibitors of ABCB1 and ABCG2, respectively. Furthermore, we discovered that the drug efflux function of both ABCB1 and ABCG2 was inhibited by sitravatinib in a concentration-dependent manner in all tested multidrug-resistant cell lines (Figure 4e).

### 2.5. Sitravatinib Does Not Alter the Protein Expression of ABCB1 or ABCG2 in Multidrug-Resistant Cancer Cells

**Figure 5:**
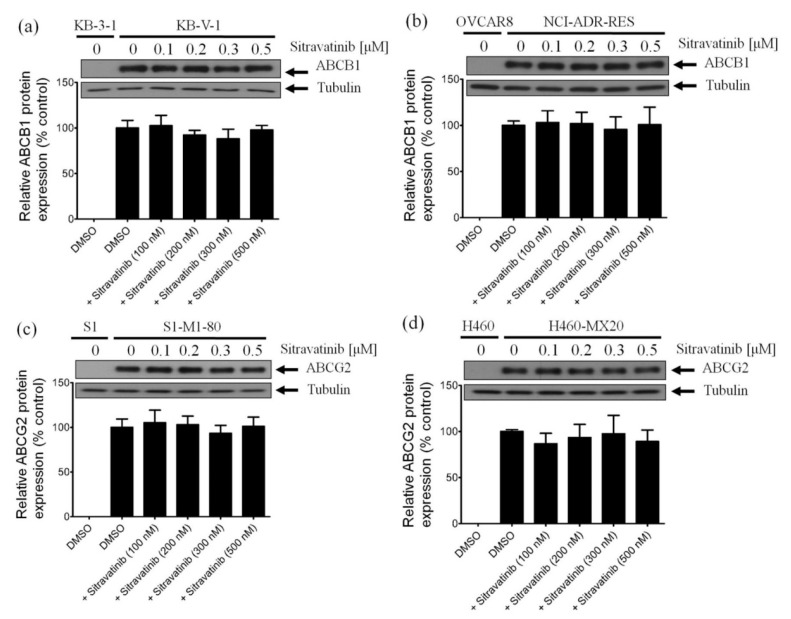
Sitravatinib has no significant effect on the protein expression of ABCB1 or ABCG2 in human multidrug-resistant cancer cells. Immunoblots (upper panel) and quantification (lower panel) of human ABCB1 protein in (**a**) KB-V-1 and (**b**) NCI-ADR-RES cancer cells or human ABCG2 protein in (**c**) S1-M1-80 and (**d**) H460-MX20 cancer cells after treatment with DMSO (vehicle control) or sitravatinib at 100 nM, 200 nM, 300 nM or 500 nM for 72 h before processed for immunoblotting as described previously [49]. α-Tubulin was used as an internal loading control. Values are presented as mean ±SD calculated from three independent experiments.

Several reports have demonstrated that in addition to inhibiting the drug efflux function of ABCB1 and ABCG2, the transient down-regulation of these transporters is one of the common alternative mechanisms in which the multidrug-resistant cancer cells can be re-sensitized to chemotherapy [23,47,48]. To this end, we examined the protein expression of ABCB1 in KB-V-1 cancer cells and ABCG2 in S1-M1-80 cancer cells by immunoblotting after treating cells with sitravatinib at various concentrations (100–500 nM) for 72 h as described in Materials and Methods. As shown in Figure 5, we were unable to find any significant changes in the protein level of ABCB1 in KB-V-1 cells (Figure 5a and Figure S1) and NCI-ADR-RES cells (Figure 5b and Figure S2) or ABCG2 in S1-M1-80 cells (Figure 5c and Figure S3) and H460-MX20 cells (Figure 5d and Figure S4), indicating that the down-regulation of ABCB1 and ABCG2 is unlikely to play a major role in the resensitization of multidrug-resistant cancer cells by sitravatinib. Our data suggest that sitravatinib re-sensitizes ABCB1- and ABCG2-overexpressing multidrug-resistant cancer cells by attenuating the drug transport function of ABCB1 and ABCG2.

### 2.6. Docking Analysis of Sitravatinib with Structures of ABCB1 and ABCG2

**Figure 6:**
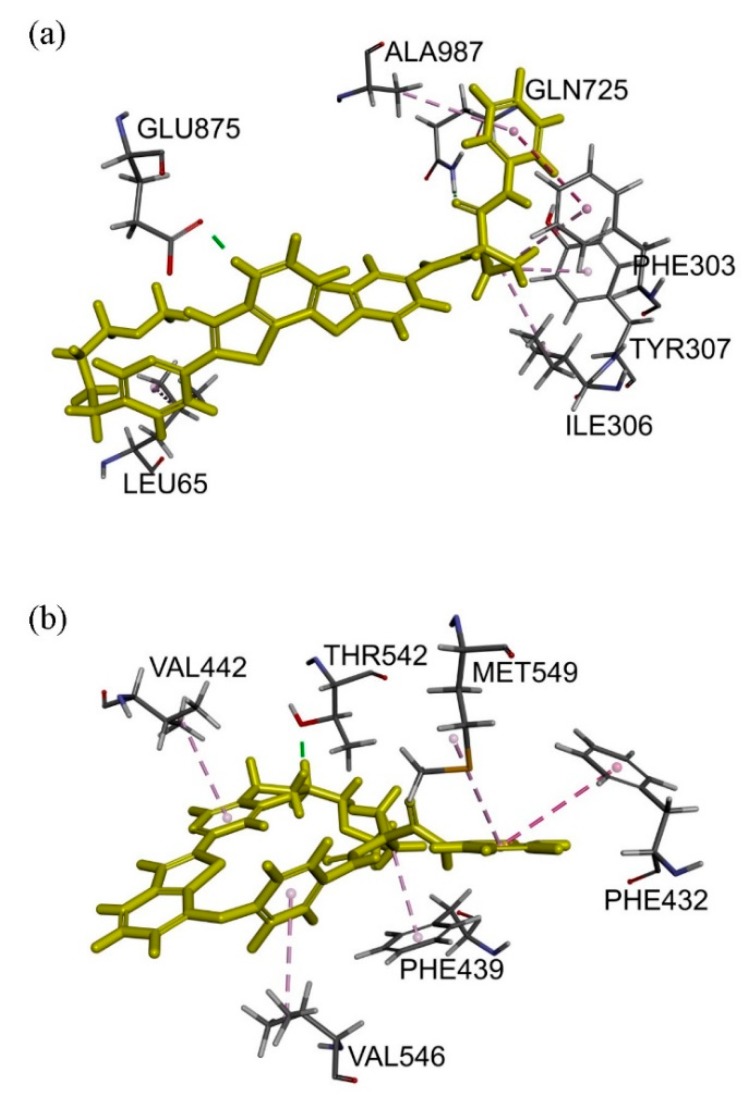
Docking of sitravatinib in the drug-binding pocket of ABCB1 and ABCG2. Binding modes of Sitravatinib with (**a**) ABCB1 and (**b**) ABCG2 protein structure (PDB: 6QEX & 5NJG) were predicted by Accelrys Discovery Studio 4.0 software as described in Materials and Methods. Sitravatinib is shown as a molecular model with highlighted yellow colour and the atoms for interacting amino acid residues were coloured carbon-grey, hydrogen-light grey, nitrogen-blue and oxygen-red. Dotted lines indicate proposed interactions.

Docking of sitravatinib with ABCB1 protein structure revealed that sitravatinib may bind to the substrate-binding sites by interacting with seven amino acid residues on transmembrane helices. Two aromatic rings, as well as cyclopropyl group on sitravatinib, were predicted to interact with PHE^303^, ILE^306^, TYR^307^, ALA^987^ and LEU^65^ via hydrophobic interaction. Hydrogen bond was found between the amide carbonyl on sitravatinib and GLN^725^ and GLU^875^ may interact with protonated nitrogen moiety on thienopyridine via both hydrogen bonding and charge interaction (Figure 6a). The binding of sitravatinib with ABCG2 protein was predicted to lie within the substrate-binding cavity between two ABCG2 monomers. One hydrogen bond was observed between residue THR^542^ and the amine on the tail of the sitravatinib structure. Four hydrophobic interactions were found between VAL^442^, VAL^546^, MET^549^ and PHE^432^ amino acid residues and pyridinyl/fluorophenyl moieties and PHE^439^ were predicted to interact with the cyclopropyl group (Figure 6b).

## 3. Discussion

The overexpression of ABCB1 and/or ABCG2 in cancer cells is known to contribute significantly to the development of multidrug resistance, an enormous challenge for scientists to overcome [3,4]. Consequently, ABCB1 and ABCG2 remain a crucial therapeutic target for improving therapeutic outcomes in cancer patients undergoing chemotherapy. Knowing that direct inhibition of the drug efflux function of ABC transporters remains the most effective approach to resensitize multidrug-resistant cancer cells to chemotherapeutic drugs, tremendous efforts have been invested into developing novel synthetic inhibitors of ABCB1 and ABCG2 [19,50]. Unfortunately, there is currently no FDA-approved therapeutic agent for the treatment of multidrug-resistant cancers. Notably, previous studies have demonstrated strong interactions between certain TKIs and ABCB1 and/or ABCG2, resulting in the resensitization of multidrug-resistant cancer cells overexpressing ABCB1 or ABCG2 [35,36,51,52]. These reports prompted us to investigate the potential chemosensitizing effect of new TKIs in multidrug-resistant cancer cells overexpressing ABCB1 or ABCG2.

Sitravatinib is an orally bioavailable TKI that targets multiple receptor tyrosine kinases [25] and potentiates immune checkpoint blockade to help cancer patients that are resistant to immune therapy [53]. In the present study, we report the potential interactions of sitravatinib with ABCB1 and ABCG2 in human multidrug-resistant cancer cells. First, the determined cytotoxicity of sitravatinib in our drug-sensitive and multidrug-resistant cell lines is comparable to the cytotoxic profile of sitravatinib reported by others in previous studies [25,53,54]. In contrast to imatinib [32] and dasatinib [34], we found that cells overexpressing ABCB1 or ABCG2 were not resistant to sitravatinib (Figure 1). More importantly, we discovered that at submicromolar concentrations, sitravatinib re-sensitized ABCB1-overexpressing cells to multiple ABCB1 substrate drugs such as paclitaxel, colchicine and vincristine (Table 2) and also re-sensitized ABCG2-overexpressing cells to several ABCG2 substrate drugs such as mitoxantrone, topotecan and SN-38 (Table 3). Similar to sitravatinib, numerous TKIs are also capable of reversing MDR mediated by ABCB1 and/or ABCG2. However, most of them are not effective towards both ABCB1 and ABCG2 at submicromolar concentrations. For instance, olmutinib selectively reverses ABCG2-mediated resistance to mitoxantrone and SN-38 at 1 and 3 micromolar concentrations [55], whereas GW583340 and GW2974 reverse ABCB1- and ABCG2-mediated drug resistance at 2.5 and 5 micromolar concentrations [56]. It is worth noting that verapamil potentiated the in vitro toxicity of vincristine irrespective of the function or expression of ABCB1 (Table 2), which is consistent with previous reports demonstrating that verapamil at nontoxic concentrations amplifying the cytotoxicity of vincristine in cancer cell lines [57,58]. Moreover, due to a trace amount of ABCG2 protein expressed in H460 cancer cells, we and others have observed evidence of chemosensitization in H460 cells by sitravatinib (Table 3) and other strong modulators of ABCG2 [59,60].

**Figure 7:**
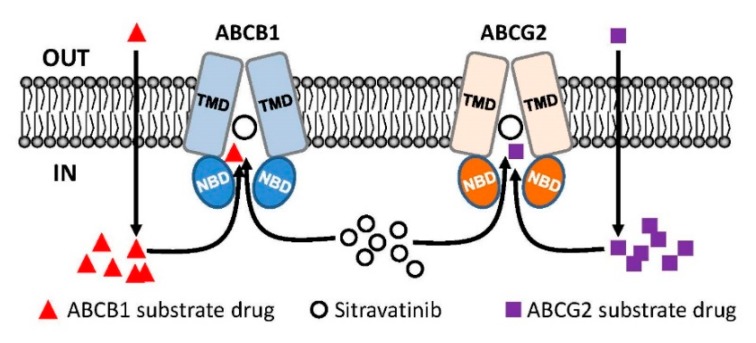
Simplified schematic diagram of sitravatinib resensitizing multidrug-resistant cancer cells by blocking the active efflux of chemotherapeutic drugs mediated by ABCB1 and ABCG2. Normally, substrate drugs of ABCB1 (red triangles) and substrate drugs of ABCG2 (purple squares) are rapidly pumped out of a multidrug-resistant cancer cell overexpressing ABCB1 (blue) or ABCG2 (orange) and consequently leading to reduced chemosensitivity. In this model, sitravatinib (white circles) competes with the binding of substrate drugs at the substrate-binding pockets of ABCB1 and ABCG2 to increase the intracellular accumulation of therapeutic drugs and eventually restoring the chemosensitivity of multidrug-resistant cancer cells.

Notably, results of sitravatinib potentiating the drug-induced apoptosis in ABCB1- and ABCG2-overexpressing cancer cells (Figure 3) indicated that sitravatinib re-sensitized multidrug-resistant cancer cells by blocking the drug efflux function of both transporters and consequently restoring the cytotoxicity of respective cytotoxic drugs and not by merely reducing the growth rate of these cancer cells. Furthermore, we found that the drug transport mediated by ABCB1 and ABCG2 was inhibited by sitravatinib in a concentration-dependent manner (Figure 4) but the protein expression of ABCB1 and ABCG2 was not significantly altered by sitravatinib in multidrug-resistant cancer cells overexpressing ABCB1 or ABCG2 (Figure 5). Nevertheless, the effect of long-term regular treatment of sitravatinib on ABCB1 and ABCG2 in human cancer cells remains to be determined. The in silico docking analysis of sitravatinib binding to the inward-open conformation of ABCB1 and ABCG2 indicated that sitravatinib can potentially interact strongly with several amino acid residues within the transmembrane regions of ABCB1 and ABCG2 (Figure 6). Our data collectively support the notion that sitravatinib reverses multidrug resistance mediated by ABCB1 and ABCG2 by binding to the substrate-binding pockets of ABCB1 and ABCG2 with relatively high affinity and competes with the binding of another therapeutic drug at the same site (Figure 7).

It is worth noting that the difficulty in the clinical applications of TKIs as chemosensitizers is mostly due to the lack of evidence of important clinical benefit or the potential unforeseen drug-drug interactions that may occur in patients [2]. Nevertheless, Moore et al. and Yang et al., reported that the efficacy of gemcitabine is enhanced significantly by erlotinib in patients with advanced pancreatic cancer [61,62]. Moreover, Geyer et al. and Cetin et al., reported that the efficacy of capecitabine was significantly improved by lapatinib in HER2-positive metastatic breast cancer patients who have previously failed conventional anticancer therapies [63,64]. More recently, encouraging results were reported in a phase I study using nilotinib as co-adjuvant treatment with doxorubicin in patients with sarcomas [65]. Collectively, these studies have shown that, in principle, a combination therapy of a cytotoxic anticancer agent and a molecularly targeted agent with a drug transporter modulating activity is a feasible therapeutic strategy against multidrug-resistant cancers. These findings are in accordance with a great deal of preclinical findings investigating the chemosensitization effect of TKIs in multidrug-resistant cancer cells overexpressing ABCB1 or ABCG2 [35,36,51]. Interestingly, like nilotinib, sitravatinib is also a TKI that re-sensitizes ABCB1- and ABCG2-overexpressing cancer cells to chemotherapeutic drugs by modulating the drug efflux function of both drug transporters [66,67,68]. Therefore, it is not inconceivable that patients with metastatic cancer exhibiting multidrug resistance may benefit from the addition of sitravatinib in combination therapy.

## 4. Materials and Methods

### 4.1. Chemicals

Sitravatinib (MGCD516) was obtained from Selleckchem (Houston, TX, USA). All other chemicals were purchased from Sigma (St. Louis, MO, USA) unless stated otherwise.

### 4.2. Cell Culture Conditions

The human epidermal cancer KB-3-1 and KB-V-1 (ABCB1-overexpressing variant) cells; the human embryonic kidney HEK293 cells, MDR19-HEK293 cells (HEK293 cells stably transfected with human ABCB1) and R482-HEK293 cells (HEK293 cells stably transfected with wild-type human ABCG2) were cultured in Dulbecco’s Modified Eagle Medium (DMEM) supplemented with 10% FCS, 2 mM l-glutamine and 100 units of penicillin/streptomycin/mL (Gibco, Invitrogen, Carlsbad, CA, USA). The human ovarian cancer OVCAR-8 cells and NCI-ADR-RES (ABCB1-overexpressing variant) cells; the human non-small cell lung cancer H460 cells and H460-MX20 (ABCG2-overexpressing variant) cells; the human colon cancer S1 cells and S1-M1-80 (ABCG2-overexpressing variant) cells were cultured in RPMI-1640 supplemented with 10% FCS, 2 mM l-glutamine and 100 units of penicillin/streptomycin/mL (Gibco, Invitrogen, Carlsbad, CA, USA). KB-V-1 cells were maintained in the presence of 1 mg/mL vinblastine [69], whereas the transfected HEK293 cells were maintained in medium containing 2 mg/mL G418 [49]. S1-M1-80 cells were cultured in the presence of 80 μM mitoxantrone [70], whereas H460-MX20 cells were cultured in the presence of 20 nM mitoxantrone [71]. Cell lines were maintained at 37 °C in 5% CO_2_ humidified air. Cells were cultured in drug-free medium for 7 days before assay.

### 4.3. Cell Viability Assay

The cytotoxicity of therapeutic drugs in the respective drug-sensitive and drug-resistant cell lines was determined using (3-(4,5-dimethylthiazol-2-yl)-2,5-diphenyltetrazolium bromide) (MTT) assay and Cell Counting Kit-8 (CCK-8) (Biotools Co., Ltd., Taipei, Taiwan) based on the method described by Ishiyama et al. [72]. Briefly, cells were plated in 96-well flat-bottom plates and allowed to attach for 24 h at 37 °C in 5% CO_2_ humidified air. Sitravatinib or other drug combination was then added to each well and incubated at 37 °C in 5% CO_2_ humidified air for an additional 72 h. The IC_50_ values were calculated using the fitted concentration-response curve of each drug regimen obtained from at least three independent experiments. The extent of chemosensitization was determined by adding a sub-toxic concentration of sitravatinib or verapamil or Ko143 to the cytotoxicity assays and the fold-reversal (FR) values were calculated as described previously [42,49].

### 4.4. Annexin V/Propidium Iodide Apoptosis Assay

To measure the effect of drug regimens on the induction of apoptotic cells, the concurrent annexin V–fluorescein isothiocyanate (FITC) and propidium iodide (PI) staining method was performed using the FITC Annexin V Apoptosis Detection Kit (BD Pharmingen, San Diego, CA, USA) according to the manufacturer protocol and as described previously [73]. Briefly, drug-sensitive and multidrug-resistant cancer cells were treated with DMSO, sitravatinib alone or with an apoptotic inducer alone or in combination as indicated for 48 h before stained with annexin V–FITC (1.25 µg/mL) and PI (0.1 mg/mL) for 15 min at room temperature. The labelled cells (10,000 cells per sample) were collected and analysed using FACScan equipped with CellQuest software (Becton-Dickinson Biosciences, San Jose, CA, USA) as described previously [24].

### 4.5. Fluorescent Drug Accumulation Assay

The intracellular accumulation of calcein, a fluorescent product of a known ABCB1 substrate calcein-AM or pheophorbide A (PhA), a known fluorescent substrate of ABCG2, was determined in the presence of DMSO (control), 5 μM of sitravatinib, 3 μM of tariquidar (a reference inhibitor of ABCB1) or 1 μM of Ko143 (a reference inhibitor of ABCG2) using a Becton-Dickinson FACSort flow cytometer as described previously [46,74]. FlowJo (Tree Star, Inc., Ashland, OR, USA) and CellQuest software were used to visualize and analyse data according to the method described by Gribar et al. [75].

### 4.6. Immunoblotting

Human cancer cells overexpressing ABCB1 or ABCG2 were treated with DMSO (control) or sitravatinib at 100 nM, 200 nM, 300 nM or 500 nM for 72 h before being harvested and subjected to SDS-polyacrylamide electrophoresis and Western blotting as described previously [49]. Primary antibodies C219 (1:3000 dilution) and BXP-21 (1:1000 dilution) were purchased from Abcam (Cambridge, MA, USA) and used to detect ABCB1 and ABCG2, respectively. Primary antibody α-tubulin (1:100,000 dilution) was used for the detection of tubulin, a positive loading control. The horseradish peroxidase-conjugated goat anti-mouse IgG (1:100,000 dilution) was purchased from Abcam (Cambridge, MA, USA) and used as secondary antibody. The enhanced chemiluminescence (ECL) kit was purchased from Merck Millipore (Billerica, MA, USA) and the signals were detected as described previously [49].

### 4.7. In Silico Docking of Sitravatinib in the Drug-Binding Pockets of ABCB1 and ABCG2

The energy of three dimensional structures of ABCB1(PDB:6QEX) [76] and ABCG2 (PDB:5NJG) [77] was minimized using Accelrys Discovery Studio 4.0 as described previously [78]. Sitravatinib structure preparation and docking were performed by the CDOCKER module of the same software.

### 4.8. Quantification and Statistical Analysis

Experimental data were determined from at least three independent experiments and the calculated IC_50_ values were presented as mean ± standard deviation (SD) unless stated otherwise. The difference between the control value or experimental value or improvement in fit was analysed by two-tailed Student’s *t*-test. The difference was considered as “statistically significant” if the probability, p, was less than 0.05 and was labelled with asterisks. GraphPad Prism software (GraphPad Software, La Jolla, CA, USA) was used for curve plotting, whereas KaleidaGraph software (Synergy Software, Reading, PA, USA) was used for statistical analysis.

## 5. Conclusions

In conclusion, our data indicated that ABCB1 and ABCG2 are unlikely to contribute significantly to the development of resistance to sitravatinib in cancer cells. Moreover, we discovered that sitravatinib could be repositioned into an effective modulator of both ABCB1 and ABCG2 to combat against multidrug resistance associated with the overexpression of ABCB1 or ABCG2 in human cancer cells. Although we cannot exclude other mechanisms that may also contribute to the resensitization of multidrug-resistant cancer cells or the potential adverse drug reactions that may occur in combination therapy [18,79,80], our results demonstrated that sitravatinib is capable of reversing multidrug resistance mediated by ABCB1 and ABCG2 at submicromolar concentrations and the concomitant administration of sitravatinib in chemotherapy warrants further investigation.

## Figures and Tables

**Table 1:** Cytotoxicity of sitravatinib in human cell lines overexpressing ABCB1 or ABCG2.

Cell Line	Type	Transporter Expressed	IC_50_ (μM) ^1^	R.F ^2^
KB-3-1	epidermal	-	2.34 ± 0.70	1.0
KB-V-1	epidermal	ABCB1	2.29 ± 0.73	1.0
OVCAR-8	ovarian	-	3.61 ± 1.64	1.0
NCI-ADR-RES	ovarian	ABCB1	5.51 ± 1.26	1.5
H460	lung	-	2.69 ± 1.07	1.0
H460-MX20	lung	ABCG2	2.24 ± 0.70	0.8
S1	colon	-	1.69 ± 0.42	1.0
S1-M1-80	colon	ABCG2	2.13 ± 0.51	1.3
pcDNA-HEK293	-	-	1.96 ± 0.50	1.0
MDR19-HEK293	-	ABCB1	2.72 ± 0.51	1.4
R482-HEK293	-	ABCG2	1.98 ± 0.45	1.0

Abbreviation: RF, resistance factor. ^1^ IC_50_ values are mean ± SD calculated from dose-response curves obtained from at least three independent experiments using cytotoxicity assay as described in Materials and Methods. ^2^ R.F values were obtained by dividing the IC_50_ value of sitravatinib in ABCB1- or ABCG2-overexpressing MDR cell lines by the IC_50_ value of sitravatinib in respective drug-sensitive parental cell lines.

**Table 2:** The effect of sitravatinib on ABCB1-mediated multidrug resistance.

Treatment	Concentration (μM)	Mean IC_50_^†^ ± SD and (FR ^‡^)	
		OVCAR-8 (Parental) [nM]	NCI-ADR-RES (Resistant) [μM]
Paclitaxel	-	3.59 ± 0.61 (1.0)	10.04 ± 1.89 (1.0)
+Sitravatinib	0.1	3.00 ± 0.50 (1.2)	6.84 ± 0.78 (1.5)
+Sitravatinib	0.2	3.13 ± 0.65 (1.1)	4.60 ± 0.47 ** (2.2)
+Sitravatinib	0.3	2.73 ± 0.44 (1.3)	1.87 ± 0.43 ** (5.4)
+Sitravatinib	0.5	2.63 ± 0.39 (1.4)	0.44 ± 0.10 *** (22.8)
+Verapamil	5.0	2.67 ± 0.57 (1.3)	0.34 ± 0.05 *** (29.5)
		[nM]	[μM]
Colchicine	-	30.91 ± 8.92 (1.0)	3.32 ± 0.71 (1.0)
+Sitravatinib	0.1	32.00 ± 9.09 (1.0)	1.84 ± 0.51 * (1.8)
+Sitravatinib	0.2	32.46 ± 9.51 (1.0)	1.03 ± 0.33 ** (3.2)
+Sitravatinib	0.3	32.66 ± 10.21 (1.0)	0.69 ± 0.09 ** (4.8)
+Sitravatinib	0.5	32.14 ± 10.01 (1.0)	0.36 ± 0.07 ** (9.2)
+Verapamil	5.0	22.86 ± 6.94 (1.4)	0.75 ± 0.14 ** (4.4)
		[nM]	[μM]
Vincristine	-	7.68 ± 1.16 (1.0)	4.29 ± 1.04 (1.0)
+Sitravatinib	0.1	7.10 ± 1.06 (1.1)	2.31 ± 0.42 * (1.9)
+Sitravatinib	0.2	6.52 ± 0.93 (1.2)	1.49 ± 0.19 * (2.9)
+Sitravatinib	0.3	6.29 ± 0.95 (1.2)	0.60 ± 0.09 ** (7.2)
+Sitravatinib	0.5	6.44 ± 1.05 (1.2)	0.15 ± 0.02 ** (28.6)
+Verapamil	5.0	1.48 ± 0.22 *** (5.2)	0.12 ± 0.02 ** (35.8)
**Treatment**	**Concentration (μM)**	**KB-3-1 (Parental) [nM]**	**KB-V-1 (Resistant) [μM]**
Paclitaxel	-	2.37 ± 0.86 (1.0)	2.76 ± 0.51 (1.0)
+Sitravatinib	0.1	2.28 ± 0.77 (1.0)	1.90 ± 0.26 (1.5)
+Sitravatinib	0.2	2.01 ± 0.69 (1.2)	1.85 ± 0.21 * (1.5)
+Sitravatinib	0.3	2.20 ± 0.76 (1.1)	0.88 ± 0.10 ** (3.1)
+Sitravatinib	0.5	2.31 ± 0.80 (1.0)	0.28 ± 0.05 ** (9.9)
+Verapamil	5.0	1.85 ± 0.62 (1.3)	66.96 ± 5.99 *** [nM] (41.2)
		[nM]	[μM]
Colchicine	-	12.50 ± 4.88 (1.0)	1.66 ± 0.12 (1.0)
+Sitravatinib	0.1	10.04 ± 3.41 (1.2)	0.43 ± 0.05 *** (3.9)
+Sitravatinib	0.2	11.85 ± 4.33 (1.1)	0.80 ± 0.02 *** (2.1)
+Sitravatinib	0.3	11.57 ± 4.38 (1.1)	0.35 ± 0.05 *** (4.7)
+Sitravatinib	0.5	11.68 ± 4.27 (1.1)	0.37 ± 0.04 *** (4.5)
+Verapamil	5.0	7.29 ± 2.19 (1.7)	0.24 ± 0.03 *** (6.9)
		[nM]	[nM]
Vincristine	-	1.23 ± 0.46 (1.0)	1479.50 ± 275.52 (1.0)
+Sitravatinib	0.1	1.19 ± 0.40 (1.0)	1207.91 ± 183.53 (1.2)
+Sitravatinib	0.2	1.31 ± 0.45 (0.9)	916.44 ± 164.61 * (1.6)
+Sitravatinib	0.3	1.38 ± 0.50 (0.9)	555.14 ± 99.66 ** (2.7)
+Sitravatinib	0.5	1.38 ± 0.45 (0.9)	118.37 ± 44.58 ** (12.5)
+Verapamil	5.0	0.24 ± 0.09 * (5.1)	25.49 ± 4.05 *** (58.0)
**Treatment**	**Concentration (μM)**	**pcDNA-HEK293 (Parental) [nM]**	**MDR19-HEK293 (Resistant) [nM]**
Paclitaxel	-	1.45 ± 0.21 (1.0)	817.35 ± 93.18 (1.0)
+Sitravatinib	0.1	1.44 ± 0.25 (1.0)	126.61 ± 18.40 *** (6.5)
+Sitravatinib	0.2	1.33 ± 0.24 (1.1)	30.55 ± 4.40 *** (26.8)
+Sitravatinib	0.3	1.44 ± 0.27 (1.0)	13.94 ± 1.62 *** (58.6)
+Sitravatinib	0.5	1.41 ± 0.27 (1.0)	7.81 ± 0.97 *** (104.65)
+Verapamil	5.0	1.02 ± 0.18 (1.4)	9.60 ± 1.52 *** (85.1)
		[nM]	[nM]
Colchicine	-	13.26 ± 3.73 (1.0)	169.27 ± 34.71 (1.0)
+Sitravatinib	0.1	11.67 ± 3.05 (1.1)	74.28 ± 10.60 * (2.3)
+Sitravatinib	0.2	11.70 ± 3.06 (1.1)	45.81 ± 7.81 ** (3.7)
+Sitravatinib	0.3	11.64 ± 3.25 (1.1)	40.74 ± 9.43 ** (4.2)
+Sitravatinib	0.5	9.38 ± 2.11 (1.4)	25.18 ± 5.72 ** (6.7)
+Verapamil	5.0	11.80 ± 3.05 (1.1)	57.09 ± 9.89 ** (3.0)
		[nM]	[nM]
Vincristine	-	1.32 ± 0.23 (1.0)	193.67 ± 31.50 (1.0)
+Sitravatinib	0.1	1.31 ± 0.30 (1.0)	54.89 ± 6.11 ** (3.5)
+Sitravatinib	0.2	1.15 ± 0.29 (1.1)	8.81 ± 1.52 *** (22.0)
+Sitravatinib	0.3	1.15 ± 0.27 (1.1)	9.53 ± 1.20 *** (20.3)
+Sitravatinib	0.5	1.04 ± 0.24 (1.3)	2.40 ± 0.43 *** (80.7)
+Verapamil	5.0	0.54 ± 0.14 ** (2.4)	1.79 ± 0.32 *** (108.2)

Abbreviation: FR, fold-reversal. ^†^ IC_50_ values are mean ±SD calculated from dose-response curves obtained from at least three independent experiments using cytotoxicity assay as described in Materials and Methods. ^‡^ FR values were calculated by dividing IC_50_ values of cells treated with a particular therapeutic drug in the absence of sitravatinib or verapamil by IC_50_ values of cells treated with the same therapeutic drug in the presence of sitravatinib or verapamil. * *p* < 0.05; ** *p* < 0.01; *** *p* < 0.001.

**Table 3:** The effect of sitravatinib on ABCG2-mediated multidrug resistance.

Treatment	Concentration (μM)	Mean IC_50_^†^ ± SD and (FR ^‡^)	
		H460 (Parental) [nM]	H460-MX20 (Resistant) [nM]
Mitoxantrone	-	57.25 ± 9.90 (1.0)	820.80 ± 118.43 (1.0)
+Sitravatinib	0.1	39.65 ± 6.73 (1.4)	592.76 ± 102.60 (1.4)
+Sitravatinib	0.2	48.17 ± 9.95 (1.2)	447.77 ± 89.18 * (1.8)
+Sitravatinib	0.3	38.98 ± 6.05 (1.5)	309.55 ± 70.08 ** (2.7)
+Sitravatinib	0.5	50.58 ± 9.62 (1.1)	238.35 ± 64.00 ** (3.4)
+Ko143	1.0	50.83 ± 10.60 (1.1)	120.15 ± 29.28 *** (6.8)
		[nM]	[nM]
Topotecan	-	67.27 ± 9.29 (1.0)	2005.50 ± 588.05 (1.0)
+Sitravatinib	0.1	53.29 ± 7.46 (1.3)	993.81 ± 312.10 (2.0)
+Sitravatinib	0.2	45.94 ± 7.52 * (1.5)	590.88 ± 206.01 * (3.4)
+Sitravatinib	0.3	49.11 ± 9.28 (1.4)	316.30 ± 114.34 ** (6.3)
+Sitravatinib	0.5	37.07 ± 6.41 ** (1.8)	333.40 ± 131.66 ** (6.0)
+Ko143	1.0	35.89 ± 6.98 ** (1.9)	75.50 ± 25.41 ** (26.6)
		[nM]	[nM]
SN-38	-	11.29 ± 1.01 (1.0)	246.22 ± 38.74 (1.0)
+Sitravatinib	0.1	9.67 ± 1.59 (1.2)	123.55 ± 24.08 ** (2.0)
+Sitravatinib	0.2	8.32 ± 1.32 * (1.4)	77.41 ± 18.10 ** (3.2)
+Sitravatinib	0.3	6.35 ± 1.04 ** (1.8)	62.12 ± 16.50 ** (4.0)
+Sitravatinib	0.5	5.43 ± 0.78 ** (2.1)	45.26 ± 14.37 ** (5.4)
+Ko143	1.0	3.27 ± 0.73 *** (3.5)	9.86 ± 3.51 *** (25.0)
**Treatment**	**Concentration (μM)**	**S1 (Parental) [nM]**	**S1-M1-80 (Resistant) [μM]**
Mitoxantrone	-	6.77 ± 1.18 (1.0)	55.46 ± 5.66 (1.0)
+Sitravatinib	0.1	7.28 ± 1.53 (0.9)	14.25 ± 1.99 *** (3.9)
+Sitravatinib	0.2	6.87 ± 1.43 (1.0)	7.13 ± 0.87 *** (7.8)
+Sitravatinib	0.3	6.01 ± 1.29 (1.1)	4.46 ± 0.53 *** (12.4)
+Sitravatinib	0.5	5.59 ± 1.18 (1.2)	1.74 ± 0.23 *** (31.9)
+Ko143	1.0	7.10 ± 1.55 (1.0)	0.53 ± 0.07 *** (104.6)
		[nM]	[nM]
Topotecan	-	35.38 ± 5.38 (1.0)	2995.95 ± 504.50 (1.0)
+Sitravatinib	0.1	34.75 ± 4.48 (1.0)	1250.80 ± 253.93 ** (2.4)
+Sitravatinib	0.2	35.25 ± 5.06 (1.0)	609.29 ± 127.64 ** (4.9)
+Sitravatinib	0.3	35.50 ± 4.72 (1.0)	422.24 ± 91.32 *** (7.1)
+Sitravatinib	0.5	40.24 ± 6.04 (0.9)	279.53 ± 63.52 *** (11.2)
+Ko143	1.0	36.39 ± 5.57 (1.0)	91.76 ± 19.15 *** (32.6)
		[nM]	[nM]
SN-38	-	4.07 ± 0.43 (1.0)	1642.50 ± 236.20 (1.0)
+Sitravatinib	0.1	4.28 ± 0.50 (1.0)	597.00 ± 128.03 ** (2.8)
+Sitravatinib	0.2	4.57 ± 0.55 (0.9)	316.89 ± 74.75 *** (5.2)
+Sitravatinib	0.3	4.57 ± 0.57 (0.9)	205.26 ± 45.33 *** (8.0)
+Sitravatinib	0.5	5.09 ± 0.68 (0.9)	121.59 ± 26.89 *** (13.5)
+Ko143	1.0	4.11 ± 0.49 (1.0)	12.39 ± 2.47 *** (132.6)
**Treatment**	**Concentration (μM)**	**pcDNA-HEK293 (Parental) [nM]**	**R482-HEK293 (Resistant) [nM]**
Mitoxantrone	-	3.86 ± 0.47 (1.0)	116.36 ± 10.39 (1.0)
+Sitravatinib	0.1	2.60 ± 0.46 * (1.5)	41.18 ± 4.02 *** (2.8)
+Sitravatinib	0.2	2.56 ± 0.44 * (1.5)	27.06 ± 3.07 *** (4.3)
+Sitravatinib	0.3	3.16 ± 0.66 (1.2)	11.44 ± 1.58 *** (10.2)
+Sitravatinib	0.5	2.66 ± 0.42 * (1.5)	12.54 ± 1.53 *** (9.3)
+Ko143	1.0	3.90 ± 0.56 (1.0)	9.71 ± 1.55 *** (12.0)
		[nM]	[nM]
Topotecan	-	26.09 ± 6.11 (1.0)	169.46 ± 22.27 (1.0)
+Sitravatinib	0.1	29.26 ± 8.03 (1.0)	71.52 ± 10.98 (1.0)
+Sitravatinib	0.2	33.39 ± 9.19 (1.0)	59.49 ± 10.19 (1.0)
+Sitravatinib	0.3	27.42 ± 7.35 (1.0)	51.96 ± 9.75 (1.0)
+Sitravatinib	0.5	29.80 ± 7.99 (1.0)	39.22 ± 8.51 (1.0)
+Ko143	1.0	28.67 ± 7.40 (1.0)	30.83 ± 8.26 (1.0)
		[nM]	[nM]
SN-38	-	4.05 ± 0.75 (1.0)	155.78 ± 13.95 (1.0)
+Sitravatinib	0.1	3.92 ± 0.83 (1.0)	23.74 ± 2.99 *** (6.6)
+Sitravatinib	0.2	4.06 ± 1.01 (1.0)	21.58 ± 2.64 *** (7.2)
+Sitravatinib	0.3	3.65 ± 0.94 (1.1)	16.17 ± 2.57 *** (9.6)
+Sitravatinib	0.5	3.39 ± 1.01 (1.2)	11.29 ± 1.79 *** (13.8)
+Ko143	1.0	4.27 ± 0.84 (0.9)	2.88 ± 0.81 *** (54.1)

Abbreviation: FR, fold-reversal. ^†^ IC_50_ values are mean ±SD calculated from dose-response curves obtained from at least three independent experiments using cytotoxicity assay as described in Materials and Methods. ^‡^ FR values were calculated by dividing IC_50_ values of cells treated with a particular therapeutic drug in the absence of sitravatinib or Ko143 by IC_50_ values of cells treated with the same therapeutic drug in the presence of sitravatinib or Ko143. * *p* < 0.05; ** *p* < 0.01; *** *p* < 0.001.

## Supplementary Materials

The following are available online at https://www.mdpi.com/2072-6694/12/1/195/s1, Figure S1: The effect of sitravatinib on the protein expression of ABCB1 in ABCB1-overexpressing human epidermal KB-V-1 cancer cells, Figure S2: The effect of sitravatinib on the protein expression of ABCB1 in ABCB1-overexpressing human ovarian NCI-ADR-RES cancer cells, Figure S3: The effect of sitravatinib on the protein expression of ABCG2 in ABCG2-overexpressing human lung H460-MX20 cancer cells, Figure S4: The effect of sitravatinib on the protein expression of ABCG2 in ABCG2-overexpressing human colon S1-M1-80 cancer cells.

## References

[1] Gottesman M., Ambudkar S.V. (2001). Overview: ABC transporters and human disease. J. Bioenergy Biomembr..

[2] Robey R.W., Pluchino K.M., Hall M.D., Fojo A.T., Bates S.E., Gottesman M.M. (2018). Revisiting the role of ABC transporters in multidrug-resistant cancer. Nat. Rev. Cancer.

[3] Gillet J.P., Gottesman M.M. (2010). Mechanisms of multidrug resistance in cancer. Methods Mol. Biol..

[4] Wu C.P., Hsieh C.H., Wu Y.S. (2011). The emergence of drug transporter-mediated multidrug resistance to cancer chemotherapy. Mol. Pharm..

[5] Szakacs G., Paterson J.K., Ludwig J.A., Booth-Genthe C., Gottesman M.M. (2006). Targeting multidrug resistance in cancer. Nat. Rev..

[6] Noguchi K., Katayama K., Sugimoto Y. (2014). Human ABC transporter ABCG2/BCRP expression in chemoresistance: basic and clinical perspectives for molecular cancer therapeutics. Pharmgenomics. Pers. Med..

[7] Kovalev A.A., Tsvetaeva D.A., Grudinskaja T.V. (2013). Role of ABC-cassette transporters (MDR1, MRP1, BCRP) in the development of primary and acquired multiple drug resistance in patients with early and metastatic breast cancer. Exp. Oncol..

[8] Schwarzenbach H. (2002). Expression of MDR1/P-glycoprotein, the multidrug resistance protein MRP, and the lung-resistance protein LRP in multiple myeloma. Med. Oncol..

[9] Tsubaki M., Satou T., Itoh T., Imano M., Komai M., Nishinobo M., Yamashita M., Yanae M., Yamazoe Y., Nishida S. (2012). Overexpression of MDR1 and survivin, and decreased Bim expression mediate multidrug-resistance in multiple myeloma cells. Leuk. Res..

[10] Turner J.G., Gump J.L., Zhang C., Cook J.M., Marchion D., Hazlehurst L., Munster P., Schell M.J., Dalton W.S., Sullivan D.M. (2006). ABCG2 expression, function, and promoter methylation in human multiple myeloma. Blood.

[11] Matthews C., Catherwood M.A., Larkin A.M., Clynes M., Morris T.C., Alexander H.D. (2006). *MDR-1*, but not *MDR-3* gene expression, is associated with unmutated IgV_H_ genes and poor prognosis chromosomal aberrations in chronic lymphocytic leukemia. Leuk. Lymphoma.

[12] Ross D.D., Karp J.E., Chen T.T., Doyle L.A. (2000). Expression of breast cancer resistance protein in blast cells from patients with acute leukemia. Blood.

[13] Steinbach D., Sell W., Voigt A., Hermann J., Zintl F., Sauerbrey A. (2002). BCRP gene expression is associated with a poor response to remission induction therapy in childhood acute myeloid leukemia. Leukemia.

[14] Uggla B., Stahl E., Wagsater D., Paul C., Karlsson M.G., Sirsjo A., Tidefelt U. (2005). BCRP mRNA expression v. clinical outcome in 40 adult AML patients. Leuk. Res..

[15] Allen J.D., Schinkel A.H. (2002). Multidrug resistance and pharmacological protection mediated by the breast cancer resistance protein (BCRP/ABCG2). Mol. Cancer.

[16] Kannan P., Telu S., Shukla S., Ambudkar S.V., Pike V.W., Halldin C., Gottesman M.M., Innis R.B., Hall M.D. (2011). The “specific” P-glycoprotein inhibitor Tariquidar is also a substrate and an inhibitor for breast cancer resistance protein (BCRP/ABCG2). ACS Chem. Neurosci..

[17] Weidner L.D., Zoghbi S.S., Lu S., Shukla S., Ambudkar S.V., Pike V.W., Mulder J., Gottesman M.M., Innis R.B., Hall M.D. (2015). The Inhibitor Ko143 Is Not Specific for ABCG2. J. Pharm. Exp..

[18] Shukla S., Wu C.P., Ambudkar S.V. (2008). Development of inhibitors of ATP-binding cassette drug transporters: Present status and challenges. Expert Opin. Drug Metab. Toxicol..

[19] Wu C.P., Calcagno A.M., Ambudkar S.V. (2008). Reversal of ABC drug transporter-mediated multidrug resistance in cancer cells: Evaluation of current strategies. Curr. Mol. Pharmacol..

[20] Shi Z., Tiwari A.K., Shukla S., Robey R.W., Singh S., Kim I.W., Bates S.E., Peng X., Abraham I., Ambudkar S.V. (2011). Sildenafil reverses ABCB1- and ABCG2-mediated chemotherapeutic drug resistance. Cancer Res..

[21] Shukla S., Chen Z.S., Ambudkar S.V. (2012). Tyrosine kinase inhibitors as modulators of ABC transporter-mediated drug resistance. Drug Resist Updates.

[22] Tiwari A.K., Sodani K., Dai C.L., Abuznait A.H., Singh S., Xiao Z.J., Patel A., Talele T.T., Fu L., Kaddoumi A. (2013). Nilotinib potentiates anticancer drug sensitivity in murine ABCB1-, ABCG2-, and ABCC10-multidrug resistance xenograft models. Cancer Lett..

[23] Wang S.Q., Liu S.T., Zhao B.X., Yang F.H., Wang Y.T., Liang Q.Y., Sun Y.B., Liu Y., Song Z.H., Cai Y. (2015). Afatinib reverses multidrug resistance in ovarian cancer via dually inhibiting ATP binding cassette subfamily B member 1. Oncotarget.

[24] Hsiao S.H., Lu Y.J., Li Y.Q., Huang Y.H., Hsieh C.H., Wu C.P. (2016). Osimertinib (AZD9291) Attenuates the Function of Multidrug Resistance-Linked ATP-Binding Cassette Transporter ABCB1 in Vitro. Mol. Pharm..

[25] Patwardhan P.P., Ivy K.S., Musi E., de Stanchina E., Schwartz G.K. (2016). Significant blockade of multiple receptor tyrosine kinases by MGCD516 (Sitravatinib), a novel small molecule inhibitor, shows potent anti-tumor activity in preclinical models of sarcoma. Oncotarget.

[26] Thomas J., Wang L., Clark R.E., Pirmohamed M. (2004). Active transport of imatinib into and out of cells: Implications for drug resistance. Blood.

[27] Breedveld P., Pluim D., Cipriani G., Wielinga P., van Tellingen O., Schinkel A.H., Schellens J.H. (2005). The effect of Bcrp1 (Abcg2) on the in vivo pharmacokinetics and brain penetration of imatinib mesylate (Gleevec): Implications for the use of breast cancer resistance protein and P-glycoprotein inhibitors to enable the brain penetration of imatinib in patients. Cancer Res..

[28] Burger H., Van Tol H., Brok M., Wiemer E.A., De Bruijn E.A., Guetens G., De Boeck G., Sparreboom A., Verweij J., Nooter K. (2005). Chronic imatinib mesylate exposure leads to reduced intracellular drug accumulation by induction of the ABCG2 (BCRP) and ABCB1 (MDR1) drug transport pumps. Cancer Biol..

[29] Nakanishi T., Shiozawa K., Hassel B.A., Ross D.D. (2006). Complex interaction of BCRP/ABCG2 and imatinib in BCR-ABL-expressing cells: BCRP-mediated resistance to imatinib is attenuated by imatinib-induced reduction of BCRP expression. Blood.

[30] Mizuno T., Fukudo M., Terada T., Kamba T., Nakamura E., Ogawa O., Inui K., Katsura T. (2012). Impact of genetic variation in breast cancer resistance protein (BCRP/ABCG2) on sunitinib pharmacokinetics. Drug Metab. Pharm..

[31] Sato H., Siddig S., Uzu M., Suzuki S., Nomura Y., Kashiba T., Gushimiyagi K., Sekine Y., Uehara T., Arano Y. (2015). Elacridar enhances the cytotoxic effects of sunitinib and prevents multidrug resistance in renal carcinoma cells. Eur. J. Pharm..

[32] Mahon F.X., Belloc F., Lagarde V., Chollet C., Moreau-Gaudry F., Reiffers J., Goldman J.M., Melo J.V. (2003). MDR1 gene overexpression confers resistance to imatinib mesylate in leukemia cell line models. Blood.

[33] Mahon F.X., Hayette S., Lagarde V., Belloc F., Turcq B., Nicolini F., Belanger C., Manley P.W., Leroy C., Etienne G. (2008). Evidence that resistance to nilotinib may be due to BCR-ABL, Pgp, or Src kinase overexpression. Cancer Res..

[34] Hiwase D.K., Saunders V., Hewett D., Frede A., Zrim S., Dang P., Eadie L., To L.B., Melo J., Kumar S. (2008). Dasatinib cellular uptake and efflux in chronic myeloid leukemia cells: Therapeutic implications. Clin. Cancer Res..

[35] Beretta G.L., Cassinelli G., Pennati M., Zuco V., Gatti L. (2017). Overcoming ABC transporter-mediated multidrug resistance: The dual role of tyrosine kinase inhibitors as multitargeting agents. Eur. J. Med. Chem..

[36] Wu S., Fu L. (2018). Tyrosine kinase inhibitors enhanced the efficacy of conventional chemotherapeutic agent in multidrug resistant cancer cells. Mol. Cancer.

[37] Kartner N., Riordan J.R., Ling V. (1983). Cell surface P-glycoprotein associated with multidrug resistance in mammalian cell lines. Science.

[38] Riordan J.R., Ling V. (1979). Purification of P-glycoprotein from plasma membrane vesicles of Chinese hamster ovary cell mutants with reduced colchicine permeability. J. Biol. Chem..

[39] Bates S.E., Medina-Perez W.Y., Kohlhagen G., Antony S., Nadjem T., Robey R.W., Pommier Y. (2004). ABCG2 mediates differential resistance to SN-38 (7-ethyl-10-hydroxycamptothecin) and homocamptothecins. J. Pharm. Exp..

[40] Miyake K., Mickley L., Litman T., Zhan Z., Robey R., Cristensen B., Brangi M., Greenberger L., Dean M., Fojo T. (1999). Molecular cloning of cDNAs which are highly overexpressed in mitoxantrone-resistant cells: Demonstration of homology to ABC transport genes. Cancer Res..

[41] Maliepaard M., Van Gastelen M.A., De Jong L.A., Pluim D., Van Waardenburg R.C., Ruevekamp-Helmers M.C., Floot B.G., Schellens J.H. (1999). Overexpression of the BCRP/MXR/ABCP gene in a topotecan-selected ovarian tumor cell line. Cancer Res..

[42] Dai C.L., Tiwari A.K., Wu C.P., Su X.D., Wang S.R., Liu D.G., Ashby C.R., Huang Y., Robey R.W., Liang Y.J. (2008). Lapatinib (Tykerb, GW572016) reverses multidrug resistance in cancer cells by inhibiting the activity of ATP-binding cassette subfamily B member 1 and G member 2. Cancer Res..

[43] Scheffer G.L., Maliepaard M., Pijnenborg A.C., Van Gastelen M.A., De Jong M.C., Schroeijers A.B., Van der Kolk D.M., Allen J.D., Ross D.D., Van der Valk P. (2000). Breast cancer resistance protein is localized at the plasma membrane in mitoxantrone- and topotecan-resistant cell lines. Cancer Res..

[44] Wu C.P., Hsiao S.H., Su C.Y., Luo S.Y., Li Y.Q., Huang Y.H., Hsieh C.H., Huang C.W. (2014). Human ATP-Binding Cassette transporters ABCB1 and ABCG2 confer resistance to CUDC-101, a multi-acting inhibitor of histone deacetylase, epidermal growth factor receptor and human epidermal growth factor receptor 2. Biochem. Pharm..

[45] Hollo Z., Homolya L., Davis C.W., Sarkadi B. (1994). Calcein accumulation as a fluorometric functional assay of the multidrug transporter. Biochim. Biophys. Acta.

[46] Robey R.W., Steadman K., Polgar O., Morisaki K., Blayney M., Mistry P., Bates S.E. (2004). Pheophorbide a is a specific probe for ABCG2 function and inhibition. Cancer Res..

[47] Cuestas M.L., Castillo A.I., Sosnik A., Mathet V.L. (2012). Downregulation of MDR1 and abcg2 genes is a mechanism of inhibition of efflux pumps mediated by polymeric amphiphiles. Bioorg. Med. Chem. Lett..

[48] Natarajan K., Bhullar J., Shukla S., Burcu M., Chen Z.S., Ambudkar S.V., Baer M.R. (2013). The Pim kinase inhibitor SGI-1776 decreases cell surface expression of P-glycoprotein (ABCB1) and breast cancer resistance protein (ABCG2) and drug transport by Pim-1-dependent and -independent mechanisms. Biochem. Pharm..

[49] Wu C.P., Shukla S., Calcagno A.M., Hall M.D., Gottesman M.M., Ambudkar S.V. (2007). Evidence for dual mode of action of a thiosemicarbazone, NSC73306: A potent substrate of the multidrug resistance linked ABCG2 transporter. Mol. Cancer.

[50] Juvale K., Wiese M. (2015). Design of inhibitors of BCRP/ABCG2. Future Med. Chem..

[51] Brozik A., Hegedus C., Erdei Z., Hegedus T., Ozvegy-Laczka C., Szakacs G., Sarkadi B. (2011). Tyrosine kinase inhibitors as modulators of ATP binding cassette multidrug transporters: Substrates, chemosensitizers or inducers of acquired multidrug resistance?. Expert Opin. Drug Metab. Toxicol..

[52] Camidge D.R., Pao W., Sequist L.V. (2014). Acquired resistance to TKIs in solid tumours: Learning from lung cancer. Nat. Rev. Clin. Oncol..

[53] Du W., Huang H., Sorrelle N., Brekken R.A. (2018). Sitravatinib potentiates immune checkpoint blockade in refractory cancer models. JCI Insight.

[54] Dolan M., Mastri M., Tracz A., Christensen J.G., Chatta G., Ebos J.M.L. (2019). Enhanced efficacy of sitravatinib in metastatic models of antiangiogenic therapy resistance. PLoS ONE.

[55] Zhang W., Fan Y.F., Cai C.Y., Wang J.Q., Teng Q.X., Lei Z.N., Zeng L., Gupta P., Chen Z.S. (2018). Olmutinib (BI1482694/HM61713), a Novel Epidermal Growth Factor Receptor Tyrosine Kinase Inhibitor, Reverses ABCG2-Mediated Multidrug Resistance in Cancer Cells. Front. Pharmacol..

[56] Sodani K., Tiwari A.K., Singh S., Patel A., Xiao Z.J., Chen J.J., Sun Y.L., Talele T.T., Chen Z.S. (2012). GW583340 and GW2974, human EGFR and HER-2 inhibitors, reverse ABCG2- and ABCB1-mediated drug resistance. Biochem. Pharm..

[57] Tsuruo T., Iida H., Naganuma K., Tsukagoshi S., Sakurai Y. (1983). Promotion by verapamil of vincristine responsiveness in tumor cell lines inherently resistant to the drug. Cancer Res..

[58] Tsuruo T., Iida H., Yamashiro M., Tsukagoshi S., Sakurai Y. (1982). Enhancement of vincristine- and adriamycin-induced cytotoxicity by verapamil in P388 leukemia and its sublines resistant to vincristine and adriamycin. Biochem. Pharm..

[59] Cai C.Y., Zhai H., Lei Z.N., Tan C.P., Chen B.L., Du Z.Y., Wang J.Q., Zhang Y.K., Wang Y.J., Gupta P. (2019). Benzoyl indoles with metabolic stability as reversal agents for ABCG2-mediated multidrug resistance. Eur. J. Med. Chem..

[60] Zhang G.N., Zhang Y.K., Wang Y.J., Gupta P., Ashby C.R., Alqahtani S., Deng T., Bates S.E., Kaddoumi A., Wurpel J.N.D. (2018). Epidermal growth factor receptor (EGFR) inhibitor PD153035 reverses ABCG2-mediated multidrug resistance in non-small cell lung cancer: In vitro and in vivo. Cancer Lett..

[61] Moore M.J., Goldstein D., Hamm J., Figer A., Hecht J.R., Gallinger S., Au H.J., Murawa P., Walde D., Wolff R.A. (2007). Erlotinib plus gemcitabine compared with gemcitabine alone in patients with advanced pancreatic cancer: A phase III trial of the National Cancer Institute of Canada Clinical Trials Group. J. Clin. Oncol..

[62] Yang Z.Y., Yuan J.Q., Di M.Y., Zheng D.Y., Chen J.Z., Ding H., Wu X.Y., Huang Y.F., Mao C., Tang J.L. (2013). Gemcitabine plus erlotinib for advanced pancreatic cancer: A systematic review with meta-analysis. PLoS ONE.

[63] Geyer C.E., Forster J., Lindquist D., Chan S., Romieu C.G., Pienkowski T., Jagiello-Gruszfeld A., Crown J., Chan A., Kaufman B. (2006). Lapatinib plus capecitabine for HER2-positive advanced breast cancer. N. Engl. J. Med..

[64] Cetin B., Benekli M., Turker I., Koral L., Ulas A., Dane F., Oksuzoglu B., Kaplan M.A., Koca D., Boruban C. (2014). Lapatinib plus capecitabine for HER2-positive advanced breast cancer: A multicentre study of Anatolian Society of Medical Oncology (ASMO). J. Chemother..

[65] Alemany R., Moura D.S., Redondo A., Martinez-Trufero J., Calabuig S., Saus C., Obrador-Hevia A., Ramos R.F., Villar V.H., Valverde C. (2018). Nilotinib as co-adjuvant treatment with doxorubicin in patients with sarcomas: A phase I trial of the Spanish Group for Research on Sarcoma. Clin. Cancer Res..

[66] Brendel C., Scharenberg C., Dohse M., Robey R.W., Bates S.E., Shukla S., Ambudkar S.V., Wang Y., Wennemuth G., Burchert A. (2007). Imatinib mesylate and nilotinib (AMN107) exhibit high-affinity interaction with ABCG2 on primitive hematopoietic stem cells. Leukemia.

[67] Tiwari A.K., Sodani K., Wang S.R., Kuang Y.H., Ashby C.R., Chen X., Chen Z.S. (2009). Nilotinib (AMN107, Tasigna) reverses multidrug resistance by inhibiting the activity of the ABCB1/Pgp and ABCG2/BCRP/MXR transporters. Biochem. Pharm..

[68] Dohse M., Scharenberg C., Shukla S., Robey R.W., Volkmann T., Deeken J.F., Brendel C., Ambudkar S.V., Neubauer A., Bates S.E. (2010). Comparison of ATP-binding cassette transporter interactions with the tyrosine kinase inhibitors imatinib, nilotinib, and dasatinib. Drug Metab. Dispos. Biol. Fate Chem..

[69] Shen D.W., Fojo A., Chin J.E., Roninson I.B., Richert N., Pastan I., Gottesman M.M. (1986). Human multidrug-resistant cell lines: Increased MDR1 expression can precede gene amplification. Science.

[70] Honjo Y., Hrycyna C.A., Yan Q.W., Medina-Perez W.Y., Robey R.W., van de Laar A., Litman T., Dean M., Bates S.E. (2001). Acquired mutations in the MXR/BCRP/ABCP gene alter substrate specificity in MXR/BCRP/ABCP-overexpressing cells. Cancer Res..

[71] Henrich C.J., Bokesch H.R., Dean M., Bates S.E., Robey R.W., Goncharova E.I., Wilson J.A., McMahon J.B. (2006). A high-throughput cell-based assay for inhibitors of ABCG2 activity. J. Biomol. Screen..

[72] Ishiyama M., Tominaga H., Shiga M., Sasamoto K., Ohkura Y., Ueno K. (1996). A combined assay of cell viability and in vitro cytotoxicity with a highly water-soluble tetrazolium salt, neutral red and crystal violet. Biol. Pharm. Bull..

[73] Anderson H.A., Maylock C.A., Williams J.A., Paweletz C.P., Shu H., Shacter E. (2003). Serum-derived protein S binds to phosphatidylserine and stimulates the phagocytosis of apoptotic cells. Nat. Immunol..

[74] Wu C.P., Hsiao S.H., Sim H.M., Luo S.Y., Tuo W.C., Cheng H.W., Li Y.Q., Huang Y.H., Ambudkar S.V. (2013). Human ABCB1 (P-glycoprotein) and ABCG2 mediate resistance to BI 2536, a potent and selective inhibitor of Polo-like kinase 1. Biochem. Pharm..

[75] Gribar J.J., Ramachandra M., Hrycyna C.A., Dey S., Ambudkar S.V. (2000). Functional characterization of glycosylation-deficient human P-glycoprotein using a vaccinia virus expression system. J. Membr. Biol..

[76] Alam A., Kowal J., Broude E., Roninson I., Locher K.P. (2019). Structural insight into substrate and inhibitor discrimination by human P-glycoprotein. Science.

[77] Taylor N.M.I., Manolaridis I., Jackson S.M., Kowal J., Stahlberg H., Locher K.P. (2017). Structure of the human multidrug transporter ABCG2. Nature.

[78] Wu C.P., Hsieh Y.J., Murakami M., Vahedi S., Hsiao S.H., Yeh N., Chou A.W., Li Y.Q., Wu Y.S., Yu J.S. (2018). Human ATP-binding cassette transporters ABCB1 and ABCG2 confer resistance to histone deacetylase 6 inhibitor ricolinostat (ACY-1215) in cancer cell lines. Biochem. Pharm..

[79] Stewart C.F., Leggas M., Schuetz J.D., Panetta J.C., Cheshire P.J., Peterson J., Daw N., Jenkins J.J., Gilbertson R., Germain G.S. (2004). Gefitinib enhances the antitumor activity and oral bioavailability of irinotecan in mice. Cancer Res..

[80] Leggas M., Panetta J.C., Zhuang Y., Schuetz J.D., Johnston B., Bai F., Sorrentino B., Zhou S., Houghton P.J., Stewart C.F. (2006). Gefitinib modulates the function of multiple ATP-binding cassette transporters in vivo. Cancer Res..

