# Supplementary material for: Sitravatinib Sensitizes ABCB1- and ABCG2-Overexpressing Multidrug-Resistant Cancer Cells to Chemotherapeutic Drugs
Source: Cancers (Basel). 2020 Jan 13;12(1):195. doi: 10.3390/cancers12010195 (PMC7017071; doi:10.3390/cancers12010195)
Supplement: Supplementary file 1 [file cancers-12-00195-s001.pdf]

Supplementary Materials: Sitravatinib Sensitizes ABCB1- and ABCG2-Overexpressing Multidrug-Resistant Cancer Cells to Chemotherapeutic Drugs

Chung-Pu Wu, Sung-Han Hsiao, Yang-Hui Huang, Lang-Cheng Hung, Yi-Jou Yu, Yu-Tzu Chang, Tai-Ho Hung and Yu-Shan Wu

IB: C219/ tubulin

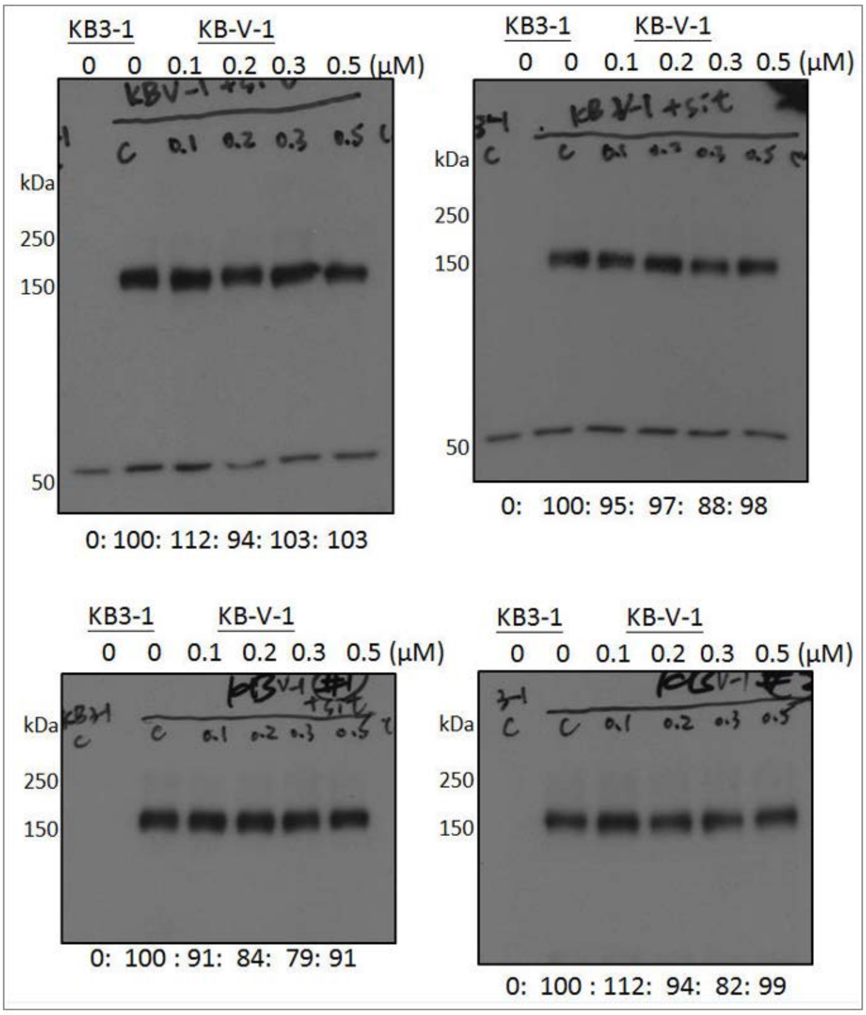

Figure 1. The effect of sitravatinib on the protein expression of ABCB1 in ABCB1-overexpressing human epidermal KB-V-1 cancer cells. KB-V-1 cancer cells were treated with DMSO (vehicle control) or sitravatinib at 100 nM, 200 nM, 300 nM or 500 nM for 72 h before processed for immunoblotting.

IB: C219/ tubulin

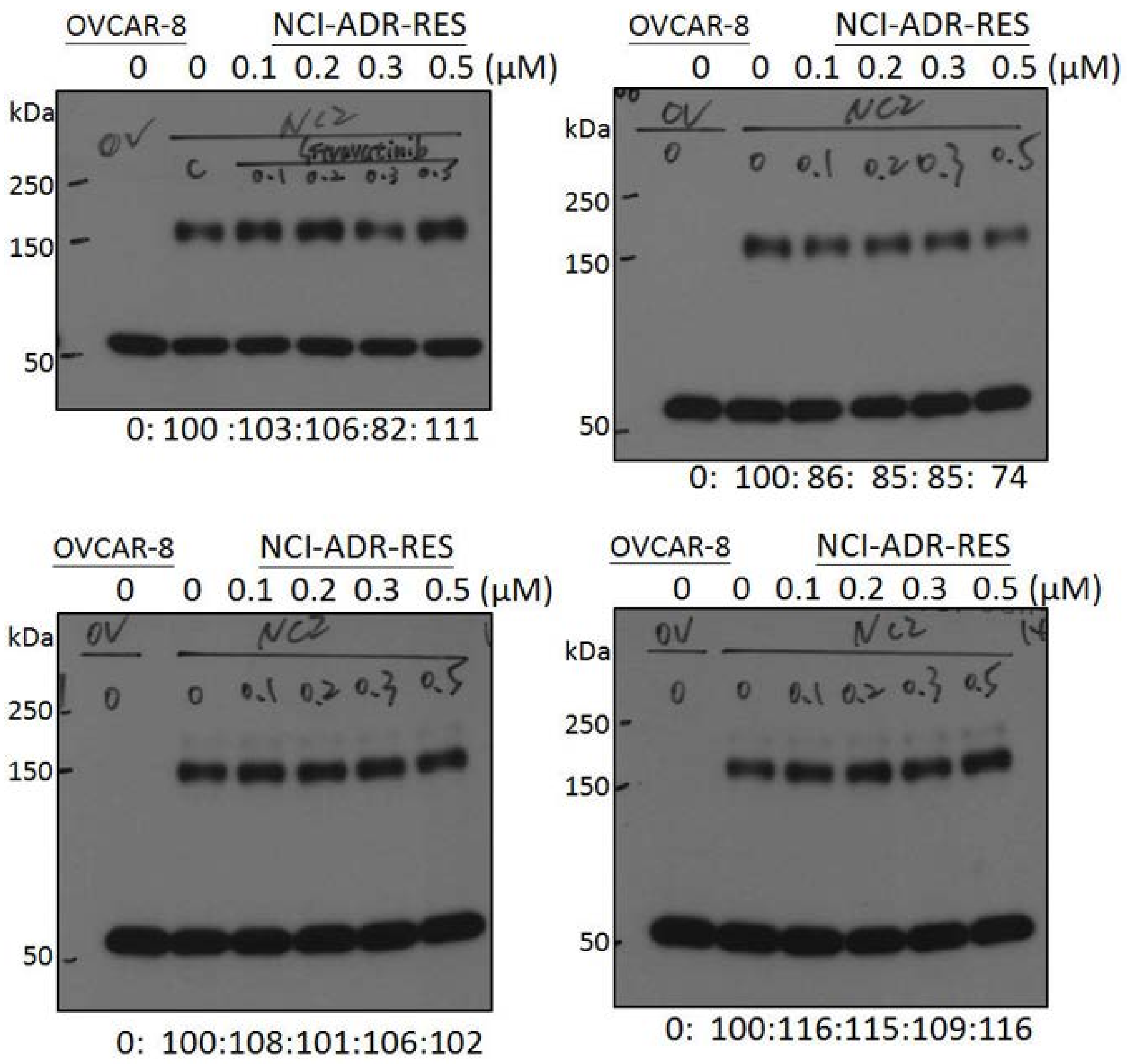

Figure S2. The effect of sitravatinib on the protein expression of ABCB1 in ABCB1-overexpressing human ovarian NCI-ADR-RES cancer cells. NCI-ADR-RES cancer cells were treated with DMSO (vehicle control) or sitravatinib at 100 nM, 200 nM, 300 nM or 500 nM for 72 h before processed for immunoblotting.

IB: BXP-21/ tubulin

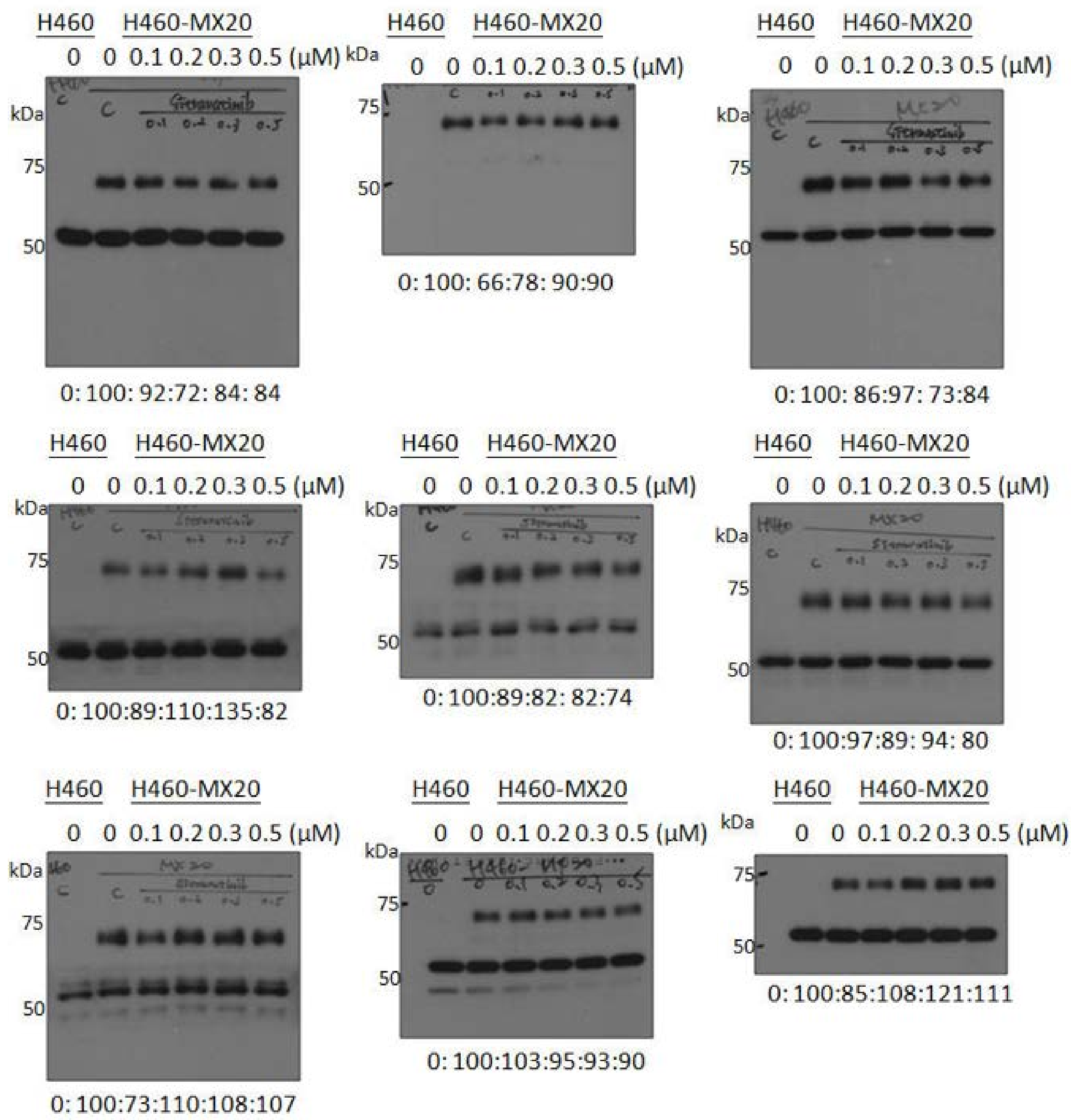

Figure 3. The effect of sitravatinib on the protein expression of ABCG2 in ABCG2-overexpressing human lung H460-MX20 cancer cells. H460-MX20 cancer cells were treated with DMSO (vehicle control) or sitravatinib at 100 nM, 200 nM, 300 nM or 500 nM for 72 h before processed for immunoblotting.

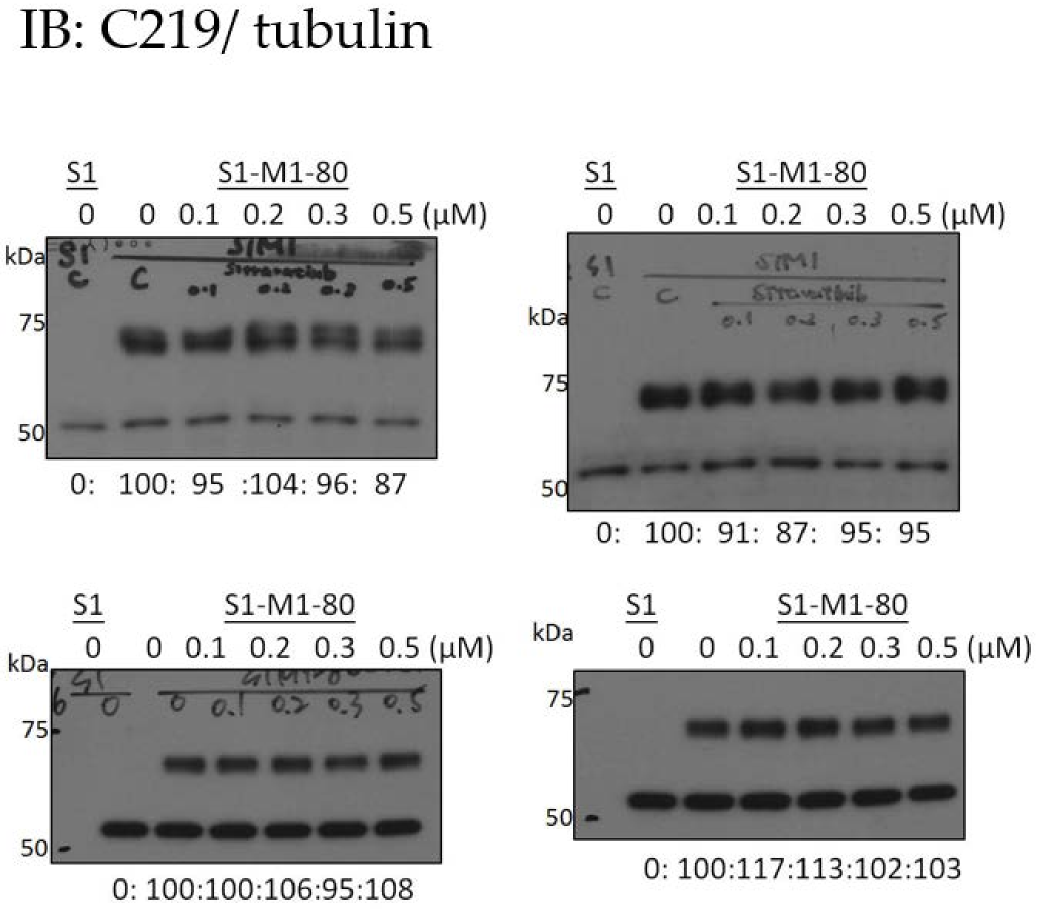

Figure 4. The effect of sitravatinib on the protein expression of ABCG2 in ABCG2-overexpressing human colon S1-M1-80 cancer cells. S1-M1-80 cancer cells were treated with DMSO (vehicle control) or sitravatinib at 100 nM, 200 nM, 300 nM or 500 nM for 72 h before processed for immunoblotting.

© 2020 by the authors. Licensee MDPI, Basel, Switzerland. This article is an open access article distributed under the terms and conditions of the Creative Commons Attribution (CC BY) license (http://creativecommons.org/licenses/by/4.0/).
